# Potentially serious incidental findings on brain and body magnetic resonance imaging of apparently asymptomatic adults: systematic review and meta-analysis

**DOI:** 10.1136/bmj.k4577

**Published:** 2018-11-22

**Authors:** Lorna M Gibson, Laura Paul, Francesca M Chappell, Malcolm Macleod, William N Whiteley, Rustam Al-Shahi Salman, Joanna M Wardlaw, Cathie L M Sudlow

**Affiliations:** 1 Usher Institute of Population Health Sciences and Informatics, University of Edinburgh, Edinburgh BioQuarter, Edinburgh EH16 4UX, UK; 2 Department of Clinical Radiology, Glasgow Royal Infirmary, Glasgow, UK; 3 Centre for Clinical Brain Sciences, University of Edinburgh, Edinburgh, UK

## Abstract

**Objectives:**

To determine prevalence and types of potentially serious incidental findings on magnetic resonance imaging (MRI) in apparently asymptomatic adults, describe factors associated with potentially serious incidental findings, and summarise information on follow-up and final diagnoses.

**Design:**

Systematic review and meta-analyses.

**Data sources:**

Citation searches of relevant articles and authors’ files in Medline and Embase (from inception to 25 April 2017).

**Review methods:**

Eligible studies included prevalence and types of incidental findings detected among apparently asymptomatic adults undergoing MRI of the brain, thorax, abdomen, or brain and body. Data on study population and methods, prevalence and types of incidental findings, and final diagnoses were extracted. Pooled prevalence was estimated by random effects meta-analysis, and heterogeneity by τ^2^ statistics.

**Main outcome measures:**

Prevalence of potentially serious incidental findings on MRI of the brain, thorax, abdomen, and brain and body.

**Results:**

Of 5905 retrieved studies, 32 (0.5%) met the inclusion criteria (n=27 643 participants). Pooled prevalence of potentially serious incidental findings was 3.9% (95% confidence interval 0.4% to 27.1%) on brain and body MRI, 1.4% (1.0% to 2.1%) on brain MRI, 1.3% (0.2% to 8.1%) on thoracic MRI, and 1.9% (0.3% to 12.0%) on abdominal MRI. Pooled prevalence rose after including incidental findings of uncertain potential seriousness (12.8% (3.9% to 34.3%), 1.7% (1.1% to 2.6%), 3.0% (0.8% to 11.3%), and 4.5% (1.5% to 12.9%), respectively). There was generally substantial heterogeneity among included studies. About half the potentially serious incidental findings were suspected malignancies (brain, 0.6% (95% confidence interval 0.4% to 0.9%); thorax, 0.6% (0.1% to 3.1%); abdomen, 1.3% (0.2% to 9.3%); brain and body, 2.3% (0.3% to 15.4%)). There were few informative data on potential sources of between-study variation or factors associated with potentially serious incidental findings. Limited data suggested that relatively few potentially serious incidental findings had serious final diagnoses (48/234, 20.5%).

**Conclusions:**

A substantial proportion of apparently asymptomatic adults will have potentially serious incidental findings on MRI, but little is known of their health consequences. Systematic, long term follow-up studies are needed to better inform on these consequences and the implications for policies on feedback of potentially serious incidental findings.

**Systematic review registration:**

Prospero CRD42016029472.

## Introduction

Magnetic resonance imaging (MRI) of the brain and body (that is, the brain, thorax, and abdomen) is increasingly used for clinical and commercial screening and for research, with several large scale, population based imaging initiatives ongoing around the world.^1^ ^2^ ^3^ ^4^ ^5^ The detection of incidental findings unrelated to the purpose of the imaging^6^ is an inevitable consequence. Clinicians and researchers should therefore anticipate incidental findings and develop appropriate policies for managing them, taking into account their expected prevalence and clinical severity.^7^ Existing data on the prevalence of incidental findings from systematic reviews of MRI of one body region,^8^ patient populations undergoing MRI,^9^ or apparently asymptomatic people imaged by another modality,^10^ are not generalisable to brain and body MRI of apparently asymptomatic people. Here, asymptomatic people are defined as community dwelling individuals not selected for imaging on the basis of symptoms, risk factors, or disease.

The clinical severity of incidental findings ranges from non-serious (eg, simple renal cyst) to potentially life threatening (eg, some malignancies), but their nature and severity are often unclear. Diagnostic radiological imaging is tailored optimally to demonstrate (or exclude) pathologies relevant to a patient’s presentation. By contrast, since incidental findings are, by definition, unrelated to the imaging’s purpose,^6^ no imaging protocol is specifically designed to optimise firm diagnoses of these findings. Further specific clinical follow-up is therefore often needed to permit final clinical diagnoses of incidental findings.

Given that knowing about clearly non-serious incidental findings would be of limited potential benefit, we focus here on potentially serious incidental findings, defined as those findings indicating the possibility of a condition which, if confirmed, would carry a real prospect of seriously threatening life span, or of having a substantial effect on major body functions or quality of life.^11^ The development of well informed approaches to the management of such potentially serious incidental findings on brain and body MRI in apparently asymptomatic adults requires data on their prevalence and types, associated factors, and the resulting final diagnoses.

We therefore aimed systematically to review studies of brain, thorax, abdomen, and brain and body MRI to determine the prevalence and types of potentially serious incidental findings among apparently asymptomatic adults, describe factors associated with such findings, and determine what is known about the follow-up and final diagnoses of people with these findings. This study was motivated by—and mainly conducted during preparations for—the ongoing UK Biobank multimodal imaging study (including brain and body MRI) of 100 000 people.^5^

## Methods

We registered the protocol for this review with Prospero,^12^ and archived data online.^13^

### Data sources

We searched Medline and Embase from inception to 25 April 2017 for references to studies in any language that reported the prevalence of incidental findings in apparently asymptomatic adults undergoing cardiac, abdominal, or brain and body MRI (supplementary methods 1). For brain MRI, we screened studies included in a published systematic review of incidental findings in apparently asymptomatic volunteers^8^ and updated the search to 25 April 2017 (supplementary methods 1). We searched authors’ files and forward and backwards citations of retrieved studies for further relevant studies.

### Study selection

One author (LMG) screened all references for potentially eligible studies. A second author (LP) independently screened a random sample of 10% of references to assess the reliability of this process. Disagreements were resolved through discussion between these authors, with arbitration by a senior author (CLMS) if necessary. We retrieved full text articles of potentially eligible studies. One author (LMG) assessed articles for inclusion, and discussed uncertainties with a senior author (CLMS).

We defined apparently asymptomatic people as those who were not selected on the basis of any symptoms, risk factors, or disease; and who attended for studies on population based research imaging, for commercial or occupational screening, or as research controls. We excluded studies of patients (that is, people selected for a study based on symptoms, risk factors, or disease; or those admitted to or attending a healthcare facility for clinical diagnostic imaging); magnetic resonance angiography that only reported vascular incidental findings (due to limited generalisability); prespecified subgroups of incidental findings (which would underestimate the prevalence of other incidental findings); children (<18 years old). Studies not published in full were also excluded. If multiple publications arose from a study, we prioritised the primary review question of prevalence, and included data from the largest cohort.

### Data extraction

One author (LMG) extracted data from all included studies on study population, study methods, and prevalence and types of all incidental findings using a pre-piloted, standardised data extraction spreadsheet. To assess the reliability of this process, a second author (LP) independently extracted data from a 10% random sample of studies. Disagreements were resolved through discussion between these authors.

#### Study and population characteristics

We extracted data on sample size, numbers of men and women, mean age and age range of participants, the country where the imaging was conducted (or, if not reported, the country of the first author’s institution), body region(s) imaged, and imaging setting (classified as either research (if participants were imaged during research studies) or non-research settings (imaging was performed in other contexts, including occupational imaging or commercial imaging)).

#### Study imaging and reporting methods of incidental findings

We extracted data on whether prevalence of incidental findings was assessed by reviewing magnetic resonance images or reports, the specialist field and number of those reporting images, blinding of reporters to information about the participants, the MRI sequences performed, and the dates when MRI was performed.

#### Data on incidental findings

We extracted data on the total number of participants with incidental findings, the total number of incidental findings, or both if available; number of participants with multiple incidental findings; prevalence of incidental findings by age, sex, imaging sequence, reporter, or any other factor assessed for association with incidental findings; and all available data on follow-up investigations, treatment, and final diagnoses for studies in which all participants with incidental findings or a specified subtype or severity of incidental findings were followed-up systematically.

#### Classification of incidental findings and final diagnoses

To determine which incidental findings were potentially serious according to our definition,^11^ we referred to a list of potentially serious and non-serious incidental findings developed by UK Biobank, based on consultations with radiologists, published literature, and the German National Cohort’s methods^14^ (supplementary methods 2). For any incidental finding not on this list, we directly applied our definition of a potentially serious incidental finding. If we had insufficient published information to apply our definition, we used study definitions of severe incidental findings, accepting that these vary somewhat between studies.^13^ Potentially serious incidental findings were classified further as suspected malignancy (eg, masses), non-malignant, or possible indicators of malignancy (incidental findings which were not masses, but could be related to malignancy, such as pleural effusions (supplementary methods 3)). We classified final diagnoses as serious if they were likely to significantly threaten lifespan or have a major effect on quality of life or major body functions, and as not serious if this was not the case. Incidental findings or final diagnoses that could not be classified were described as “indeterminate.”

### Risk of bias assessment

In the absence of a validated quality assessment tool for studies of the prevalence of incidental findings, we extracted data on study characteristics that might affect risk of bias (sample selection methods, blinding of reporters to information about the participants, the specialty and number of image readers, and whether data on incidental findings were generated from reads of images or extracted from reports), and planned to consider their potential influence on the results through a series of subgroup analyses.

### Data synthesis

We meta-analysed studies with a random effects model,^15^ using maximum likelihood estimation methods^16^ and modelling within-study variance as binomial, to calculate pooled prevalence of potentially serious incidental findings and of suspected malignant incidental findings, separately for brain, thorax, abdomen, and brain and body MRI. For the pooled estimates, we calculated both 95% confidence intervals and 95% prediction intervals; prediction intervals indicate the range of true prevalence values expected in future studies.^17^ We used *t* scores (rather than the usual *z* score) to calculate 95% confidence intervals, generating conservative estimates and allowing comparison with our prediction intervals (which also use *t* scores). Region specific data from studies of brain and body MRI were included in the brain, thoracic, and abdominal MRI meta-analyses. We derived data on thoracic incidental findings from studies of either cardiac or brain and body MRI or both. To obtain upper estimates of the prevalence of potentially serious incidental findings and of suspected malignant incidental findings, we performed sensitivity meta-analyses by adding the indeterminate incidental findings to the potentially serious incidental findings, and possible indicators of malignancy to the suspected malignant incidental findings. We calculated 95% confidence intervals for individual studies’ prevalence estimates using Clopper Pearson exact methods. 

We assessed statistical heterogeneity using τ^2^ statistics, which provide a logit scale measure of between-study variance, represented in a more readily interpretable way by the 95% prediction intervals. All study level characteristics were initially considered as potential candidates for subgroup analyses, to explore reasons for heterogeneity of the prevalence of potentially serious incidental findings. However, we chose not to conduct subgroup analyses that were likely to be uninformative (eg, owing to missing data for a large proportion of studies or substantial imbalance in subgroup sizes). We performed subgroup analyses by including study characteristics as covariates in the meta-analyses.^18^ Formal statistical tests for possible publication bias were not performed because their application is limited in meta-analyses where outcome is expressed as a proportion.^19^ ^20^ We also decided not to conduct formal meta-analysis of data on the percentage of potentially serious incidental findings that resulted in serious final diagnoses (that is, the positive predictive value of potentially serious incidental findings), to avoid undue emphasis on the limited data available. Instead, we described available findings and calculated a rough estimate of this percentage by summing numerators and denominators across the few studies with relevant data.

We used Microsoft Excel 2013 for descriptive statistical analyses, StatsDirect 3.0.177 for calculating 95% confidence intervals for individual studies, and SAS 9.4 PROC NLMIXED (www.sas.com) for meta-analyses. 

### Patient involvement

Patients were not involved in the development or design of this study. The results of this study will be disseminated to the public by the investigators where possible.

## Results

Two authors agreed on 99% of the duplicate screened reference selections, and 100% of the duplicate extracted data.

### Included studies

Of 5905 retrieved studies, 32^21^ ^22^ ^23^ ^24^ ^25^ ^26^ ^27^ ^28^ ^29^ ^30^ ^31^ ^32^ ^33^ ^34^ ^35^ ^36^ ^37^ ^38^ ^39^ ^40^ ^41^ ^42^ ^43^ ^44^ ^45^ ^46^ ^47^ ^48^ ^49^ ^50^ ^51^ ^52^ met the inclusion criteria. These 32 studies included 27 643 participants (range 2-5800 participants, mean/median age range 21-75 years, 14 037/27 643 (50.8%) men) imaged between 1985 and 2016 (supplementary figure 1, supplementary table 1). The included studies comprised eight of brain and body MRI,^21^ ^22^ ^23^ ^24^ ^25^ ^26^ ^27^ ^28^ 22 of brain MRI,^29^ ^30^ ^31^ ^32^ ^33^ ^34^ ^35^ ^36^ ^37^ ^38^ ^39^ ^40^ ^41^ ^42^ ^43^ ^44^ ^45^ ^46^ ^47^ ^48^ ^49^ ^50^ and two of cardiac MRI.^51^ ^52^ No abdomen only studies were identified (supplementary table 1). 

Studies were performed in Europe (20 studies,^21^ ^22^ ^23^ ^24^ ^25^ ^27^ ^28^ ^29^ ^31^ ^34^ ^36^ ^37^ ^39^ ^40^ ^41^ ^43^ ^44^ ^47^ ^48^ ^52^ 17 702 participants), North America (six,^30^ ^35^ ^38^ ^46^ ^50^ ^51^ 5789), Asia (four,^26^ ^32^ ^33^ ^45^ 3576), and Australia (two,^42^ ^49^ 576; supplementary table 1). All but three studies assessed images for incidental findings; one assessed imaging reports,^49^ and two did not report on this.^32^ ^47^ All studies involved radiologists, except for one in which a cardiologist reported incidental findings on cardiac MRI (supplementary table 1).^52^ In two studies, radiologists confirmed incidental findings detected by trained readers (defined as researchers with training to doctor of medicine level or training in neuropsychiatry) in one^29^ and MRI scan operators (not further defined) in the other.^45^

### Imaging sequences

The vast majority of participants were imaged by scanners of 1.5 T or less (19 studies, 23 809/27 643 (86.1%) participants).^21^ ^22^ ^23^ ^24^ ^25^ ^27^ ^29^ ^30^ ^31^ ^32^ ^33^ ^34^ ^36^ ^37^ ^41^ ^42^ ^48^ ^49^ ^51^ However, seven studies (1556 (5.6%) participants) used 3.0 T scanners,^26^ ^28^ ^39^ ^40^ ^43^ ^50^ ^52^ two (370 (1.3%)) used 1.5 T in some participants and 3.0 T in others,^44^ ^45^ and four (1908 (6.9%)) did not report magnet strength (supplementary table 2).^35^ ^38^ ^46^ ^47^ All but three brain MRI studies^23^ ^36^ ^47^ used T1 weighted imaging. One study used T1 weighted imaging in an unknown subset of participants.^40^ Of 10 thoracic MRI studies, eight used non-contrast whole thorax imaging (n=4817),^21^ ^22^ ^23^ ^24^ ^25^ ^26^ ^27^ ^28^ and five used cardiac specific sequences (n=4099).^21^ ^22^ ^24^ ^51^ ^52^ All abdominal MRI studies used T1 weighted imaging (supplementary table 2).

### Risk of bias assessment

Only one study appeared to have imaged an unselected, random population sample (n=2500).^21^ Most of the remaining studies imaged selected samples or did not clearly report sampling methods. At least one radiologist reported all images in almost all studies; 14 studies (8199 (29.7%) participants)^21^ ^22^ ^23^ ^24^ ^26^ ^27^ ^28^ ^33^ ^34^ ^37^ ^43^ ^46^ ^48^ ^51^ had more than one reader for each set of images (supplementary table 1). Data on blinding of readers to participants’ characteristics were incomplete, with only 16 studies (19 617 (71.0%) participants)^21^ ^23^ ^24^ ^27^ ^29^ ^30^ ^31^ ^34^ ^36^ ^37^ ^38^ ^41^ ^44^ ^45^ ^48^ ^49^ clearly reporting blinding of image readers to participant characteristics (supplementary table 1). We saw no direct within-study comparisons between radiologist and non-radiologist readers, between single and multiple readers, or between blinding and non-blinding of readers to participants’ characteristics, to reliably inform on any potential biases such methods might have on the prevalence of potentially serious incidental findings.

### Prevalence and types of potentially serious incidental findings

**Fig 1:**
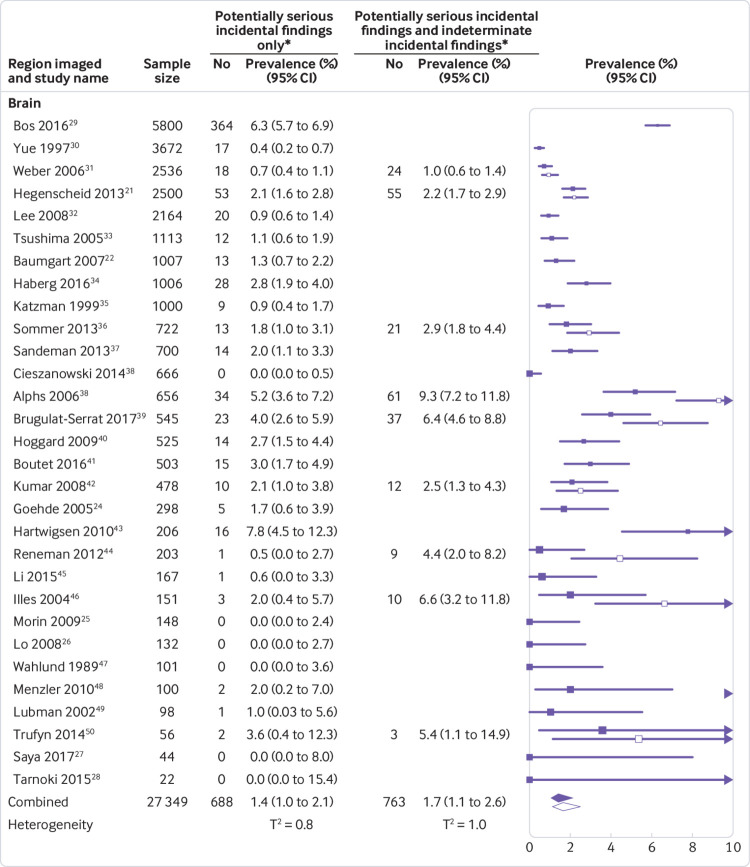
Forest plots of prevalence per study and pooled prevalence estimates of potentially serious incidental findings and of potentially serious incidental findings plus indeterminate incidental findings, detected on magnetic resonance imaging (MRI) of the brain. T^2^=estimate of between-study variance on the logit scale (0 indicates no variance, increasing values indicate increasing heterogeneity). Solid squares and diamonds=point prevalence per study and pooled prevalence estimate of potentially serious incidental findings on brain MRI; white squares and diamonds=sensitivity analyses that include incidental findings classified as indeterminate in the point prevalence per study and pooled prevalence estimate of potentially serious incidental findings on brain MRI. Details of types and numbers of potentially serious incidental findings are provided in figure 3 and supplementary table 4a, while details of indeterminate findings are available online.^13^ *138 vascular incidental findings detected in six studies that used MR angiography^24^ ^31^ ^32^ ^33^ ^34^ ^38^ were excluded from pooled analyses

**Fig 2:**
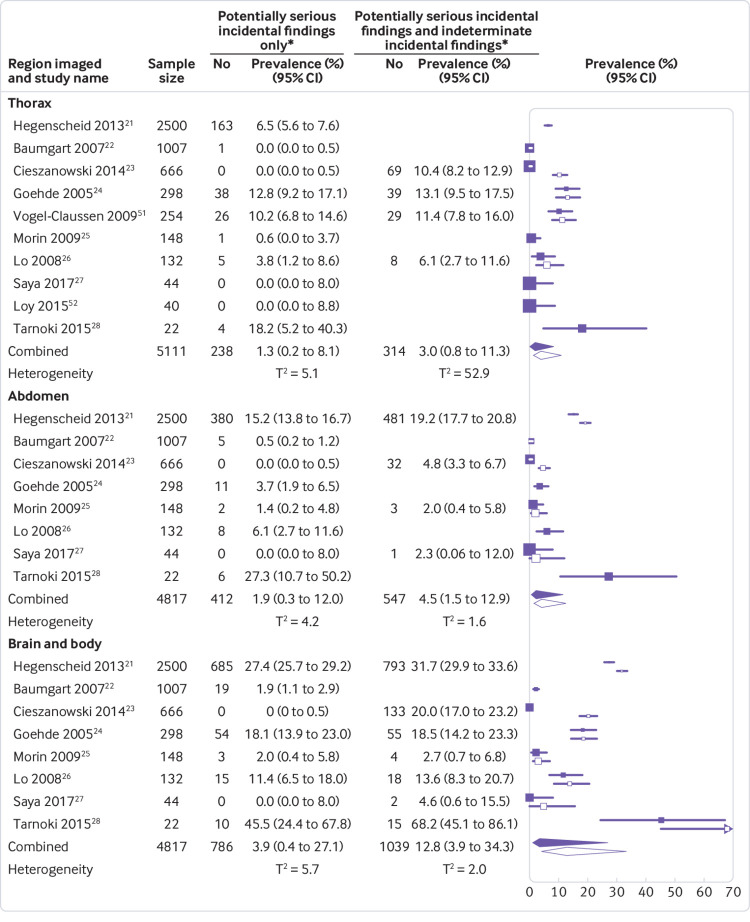
Forest plots of prevalence per study and pooled prevalence estimates of potentially serious incidental findings and of potentially serious incidental findings plus indeterminate incidental findings, detected on magnetic resonance imaging (MRI) of the thorax, abdomen, and brain and body (that is, brain, thorax, and abdomen combined). T^2^=estimate of between-study variance on the logit scale (0 indicates no variance, increasing values indicate increasing heterogeneity). Solid squares and diamonds=point prevalence per study and pooled prevalence estimate of potentially serious incidental findings on thoracic, abdominal, and brain and body MRI; white squares and diamonds=sensitivity analyses that include incidental findings classified as indeterminate in the point prevalence per study and pooled prevalence estimate of potentially serious incidental findings on thoracic, abdominal, and brain and body MRI. Details of types and numbers of potentially serious incidental findings are provided in figure 3 and supplementary tables 4b-c, while details of indeterminate findings are available online.^13^ *200 incidental findings detected in studies that used specialist imaging sequences (97 breast lesions in a study including magnetic resonance mammography,^21^ 87 colonic polyps in two studies including magnetic resonance colonography,^22^ ^24^ 15 vascular findings such as stenosis or plaque in four studies including magnetic resonance angiography,^21^ ^22^ ^24^ ^28^ and one myocardial infarction in a study including post-contrast cardiac imaging^24^) were excluded from pooled analyses

Although 14 studies^21^ ^24^ ^25^ ^27^ ^31^ ^32^ ^34^ ^36^ ^37^ ^38^ ^41^ ^43^ ^50^ ^51^ reported data on multiple incidental findings per participant, none provided the number of participants with more than one potentially serious incidental finding, or data to enable calculations of this number. We therefore based prevalence estimates on the assumption that no participant had more than one potentially serious incidental finding, recognising that very few participants may have more than one. The pooled prevalences of potentially serious incidental findings on brain, thoracic, abdominal, and brain and body MRI were 1.4% (95% confidence interval 1.0% to 2.1%), 1.3% (0.2% to 8.1%), 1.9% (0.3% to 12.0%), and 3.9% (0.4% to 27.1%), respectively. When indeterminate incidental findings were included, pooled prevalence estimates increased to 1.7% (1.1% to 2.6%), 3.0% (0.8% to 11.3%), 4.5% (1.5% to 12.9%), and 12.8% (3.9% to 34.3%), respectively. Study specific prevalence estimates ranged widely, with correspondingly wide prediction intervals and τ^2^ values ranging from 0.8 to 5.7 (indicative of substantial variance between studies; fig 1 and fig 2, supplementary figure 2, and supplementary table 3).

**Fig 3:**
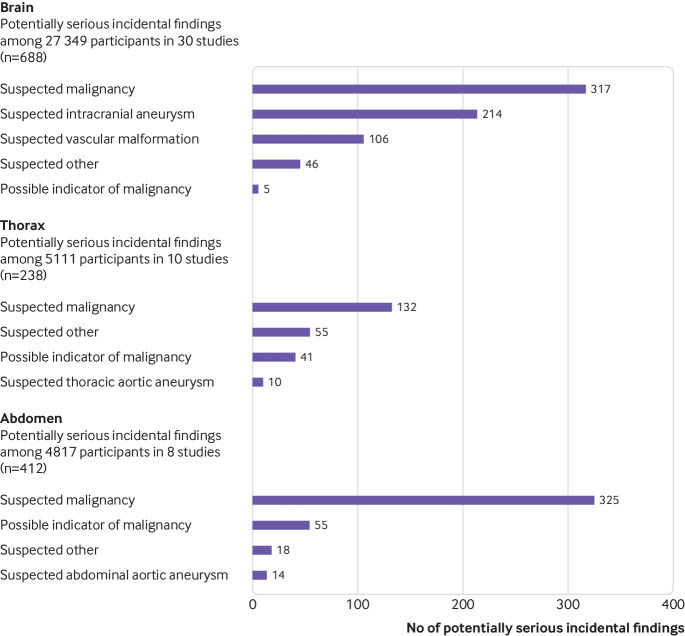
Numbers and types of potentially serious incidental findings on magnetic resonance imaging (MRI), by body region. Further details of the types of potentially serious incidental findings are provided in supplementary tables 4a-c. Potentially serious incidental findings were further classified as suspected malignancy (eg, masses), possible indicators of malignancy (incidental findings that were not masses, but could be related to malignancy, such as pleural effusions), or non-malignant (supplementary methods 3). In this figure, potentially serious incidental findings that were not suspected malignancies, possible indicators of malignancies, or suspected vascular findings were grouped as “suspected other”

Across body regions, suspected malignancies were the most common types of potentially serious incidental findings (accounting for roughly half of all such findings), with vascular findings also common on brain MRI (fig 3 and supplementary tables 4a-c). Pooled prevalence of potentially serious incidental findings suspected to be malignant were 0.6% (95% confidence interval 0.4% to 0.9%) on brain MRI, 0.6% (0.1% to 3.1%) on thorax MRI, 1.3% (0.2% to 9.3%) on abdomen MRI, and 2.3% (0.3% to 15.4%) on brain and body MRI. After possible indicators of malignancy were included, these prevalences were 0.6% (0.4% to 0.9%), 1.0% (0.2% to 5.4%), 1.6% (0.2% to 10.9%), and 3.0% (0.4% to 20.4%), respectively (supplementary figure 2).

### Subgroup analyses

Examination of the available data (supplementary tables 1 and 2) showed that several potential subgroup analyses would be uninformative owing to imbalanced subgroups or non-reporting of the relevant data for a large subset of studies. One or both of these reasons precluded subgroup analyses with respect to magnet strength (almost all 1.5 T), contrast use (incomplete data), data source (almost all studies used images rather than reports of these), image reader specialty (almost all studies had reporting by radiologists), and sample selection method (only one study randomly selected participants).^21^ We did not conduct subgroup analyses by age or sex (because we did not have individual participant data to allow meaningful comparisons), study country (because there was no clear a priori reason for variation in prevalences of potentially serious incidental findings by country), or body region (because studies of brain and body MRI contributed data on different body regions from the same participants, violating the assumption that data within different subgroups are independent). We conducted subgroup analyses for brain and body MRI and region specific MRI for imaging setting (research *v* non-research) and for factors that might inform on risks of bias (blinding of readers to participant characteristics and number of image readers) where sufficient data allowed. There was no evidence of any clinically meaningful or statistically significant difference in prevalence of potentially serious incidental findings after the inclusion of subgroups as covariates (supplementary figures 3a-i, supplementary table 5).

### Study specific reports of factors associated with potentially serious incidental findings

Eight studies reported factors associated with potentially serious incidental findings,^25^ ^27^ ^29^ ^30^ ^34^ ^36^ ^39^ ^42^ while a further five reported factors associated with incidental findings requiring follow-up, which we considered an approximate proxy for potentially serious incidental findings (supplementary tables 6a-c).^33^ ^37^ ^43^ ^46^ ^52^ Two studies found significant associations between incidental findings requiring follow-up and increasing age,^43^ ^46^ while another two found a consistently higher prevalence of incidental findings requiring follow-up^33^ and cavernomata^39^ in older age groups, although the increased prevalence was not statistically significant (supplementary table 6a). We saw no clear variation in prevalence of potentially serious incidental findings by sex (supplementary table 6b). Too few data were available on other factors (including medical history, symptoms, lifestyle factors, and genetics) to show any clear associations with potentially serious incidental findings (supplementary table 6c). No data were available on the associations between imaging sequence or reporter specialty with prevalence of potentially serious incidental findings.

### Follow-up and final diagnoses

Only five studies systematically followed up and reported data on the final clinical diagnoses of selected subsets of participants with incidental findings (a total of 234 participants followed up), representing 1.4% to 18.2% of all imaged participants in these studies (table 1).^25^ ^26^ ^27^ ^29^ ^37^ Summing arithmetically across these studies, only 48 of these 234 participants (that is, about one fifth) had clinically serious final diagnoses (although half had indeterminate final diagnoses, mostly from one study of brain MRI,^29^ in which participants were managed under “wait and see” policies). No study reported follow-up in a manner that enabled enumeration of the clinical assessments (eg, further imaging examinations, specialty referrals, biopsies) performed to clarify final diagnoses.

**Table 1:** Methods used to follow up 234 people with potentially serious incidental findings and severity of final diagnoses

Study variables				Methods of follow-up of incidental findings				Severity of final diagnoses (No)		
First author surname and year of publication	Imaged body regions	No of participants followed up/total No imaged (%)		Subset of participants followed up*	Data type (source)	Duration of follow-up		Serious	Non-serious	Indeterminate
Bos 2016^29^	Brain	188/5800 (3.2)		Those with an incidental finding who were referred to specialists†	Clinical management (medical records)	Until last clinical follow-up or death		39	34	115
Sandeman 2013^37^	Brain	10/700 (1.4)		Those with an incidental finding who were referred to family doctors‡	Resulting action (medical records)	Not specified		5	5	0
Morin 2009^25^	Brain and body	5/148 (3.4)		Those with highly significant findings§	Investigations and treatments (contact with general practitioner or participant)	Not specified		0	3	2
Lo 2008^26^	Brain and body	24/132 (18.2)		Those with an incidental finding that required further investigation¶	Not specified	Not specified		4	20	0
Saya 2017^27^	Brain and body	7/44 (15.9)		Those with incidental findings deemed to require follow-up**	Investigations (not specified**)**	Not specified		0	7	0
Total No (% of 234 followed up)				Not applicable	Not applicable	Not applicable		48 (20.5)	69 (29.5)	117 (50)

* This group could be considered a study specific proxy for potentially serious incidental findings but is not identical to the consistent definition applied in the present study. Hence study specific numbers in this table differ from study specific numbers of potentially serious incidental findings in meta-analyses.

† Decision for referral depended on the incidental finding and consultation with clinicians.

‡ Decision for referral depended on discussion between radiologists and a geriatrician and other clinicians as necessary.

§ Highly significant findings were defined as requiring prompt medical follow-up, such as indeterminate masses in solid organs, enlarged lymph nodes and ovarian masses or cysts, as judged by consensus of two radiologists. Participants’ family doctors were informed of the finding.

¶ Definition of incidental findings requiring further investigation, or processes for judging this are not reported.

** As determined by study radiologists, follow-up was discussed by a multidisciplinary team including principal investigators, radiologists, and other study staff (not otherwise specified).

## Discussion

### Principal findings

We performed meta-analyses of published studies of the prevalence of potentially serious incidental findings among apparently asymptomatic adults undergoing MRI of the brain, thorax, abdomen, or brain and body. The pooled prevalence of potentially serious incidental findings was 3.9% on brain and body MRI (1.4% brain, 1.3% thorax, and 1.9% abdomen). After including incidental findings of uncertain potential seriousness, pooled prevalence increased to 12.8% (1.7% brain, 3.0% thorax, and 4.5% abdomen). We saw wide variation among studies in their prevalence estimates, probably reflecting variation between studies in participants’ characteristics, imaging setting, sample selection methods, and methods of detecting incidental findings, as well as the challenges of applying a consistent definition of potentially serious incidental findings to the available descriptions of incidental findings in published papers. Suspected malignant incidental findings accounted for around half of all potentially serious incidental findings on brain, thoracic, abdominal, and brain and body MRI (0.6%, 0.6%, 1.3%, and 2.3% respectively). The very limited systematic follow-up data available (mainly from brain MRI studies) show that only about one fifth of people with a potentially serious incidental finding had a serious final clinical diagnosis.

### Strengths and limitations of this study

By including all identified published data on the prevalence of potentially serious incidental findings on brain, thoracic, abdominal, and brain and body MRI, and by applying a consistent definition of potentially serious incidental findings across studies, we have provided important data on the prevalence of those incidental findings that may have an important impact on health. This review includes data on potentially serious incidental findings from different body regions, enabling comparisons of prevalence between regions. As such, our results can inform people undergoing (or staff conducting) brain and body MRI or region specific MRI in apparently asymptomatic adult volunteers. As most studies selected apparently asymptomatic populations, our results can apply directly to imaging performed for research and non-research settings such as screening.

Although we have not shown evidence of a statistically significant difference in the prevalence of potentially serious incidental findings between body regions, the pooled point prevalences were generally higher on abdomen MRI and on brain and body MRI than on brain or thorax MRI, particularly when indeterminate findings were included in sensitivity analyses. This pattern is biologically plausible and has been seen in data from primary studies.^21^ ^25^ ^26^ ^28^ ^53^ The heterogeneity between included studies, relative rarity of potentially serious incidental findings, methods of meta-analyses, and conservative calculation of confidence intervals could have obscured true differences in the prevalence of potentially serious incidental findings between regions. Results on incidental findings from ongoing imaging studies based on large populations (including the UK Biobank imaging substudy, which by late October 2018 had imaged more than 30 000 of an intended 100 000 participants) should be able to confirm or refute this pattern in future.^5^ ^14^ ^54^ ^55^

We found no evidence of any meaningful differences in the prevalence of potentially serious incidental findings between studies conducted in research or imaging settings for any body region, or between studies using readers blinded to participant characteristics versus not blinded or not stated, or for brain MRI studies using one reader versus more than one reader. Further subgroup analyses that could inform on factors influencing variation in prevalence in different body regions were limited, as data on relevant variables were either lacking for a large subset of studies, or resulted in very imbalanced subgroups.

Data were included in the review after screening and extraction by one author, rather than multiple authors. Although this method could limit the accuracy of the data extraction, it is unlikely to have substantially affected our results given the good agreement with a second reviewer on a 10% subset of the studies. Owing to the lack of data on participants with more than one potentially serious incidental finding, prevalence estimates were based on the assumption that only one potentially serious incidental finding occurs per participant. However, it is unlikely that a substantial proportion of participants had more than one potentially serious incidental finding. The prevalence of incidental findings deemed “potentially serious” could vary with opinion and over time as evidence of their natural history accrues.

We could not explore the influence of technical imaging factors (eg, image resolution, magnet strength) on the prevalence of potentially serious incidental findings, because of limited data availability and reporting consistency, but these are unlikely to substantially influence the detection of the most common potentially serious incidental findings (suspected malignancies and aneurysms). The vast majority of included studies involved systematic radiologist reviews of images to detect incidental findings. No study directly compared radiologist readers with non-radiologist readers, although other policies to detect incidental findings might produce different results, such as radiographers flagging any concerning examinations for a radiologist to review.^55^

### Comparison with other studies

A recent umbrella review of incidental findings arising from a range of imaging modalities (including MRI) found no existing systematic reviews of the prevalence of incidental findings in apparently asymptomatic volunteers on cardiac, abdominal, or brain and body MRI for comparison with our findings.^56^

Our update of an existing systematic review by Morris and colleagues^8^ of incidental findings on brain MRI resulted in similar prevalence of suspected malignant incidental findings. In the recent umbrella review mentioned above, researchers reported a prevalence of incidental findings on brain MRI of 22% (95% confidence interval 14% to 31%), about 10 times higher than our pooled prevalence estimate for brain MRI.^8^ ^56^ ^57^ Most of this difference is likely to be due to the umbrella review’s inclusion of all reported incidental findings, regardless of their potential clinical significance, whereas we focused on potentially serious incidental findings. Some of the difference might also be due to different study inclusion criteria (reflecting the different focus of the umbrella review, which had broader inclusion criteria, including studies of patients as well as apparently asymptomatic people), as well as a difference in meta-analytical methods. Prevalence data, as proportions, will have a binomial distribution. The umbrella review used an arcsine transformation in its analyses of prevalence data, which avoids the challenge of directly modelling binomial data, whereas we used an exact method, which models the within-study variance as binomial to generate unbiased estimates.^16^

The recent umbrella review also reported far more final diagnosis data from studies derived from Morris and colleagues than we have in the present study.^56^ To calculate the proportion of incidental findings resulting in known final diagnoses, the participants who form the denominator should all undergo systematic follow-up in order to generate an accurate numerator. We therefore scrutinised reports of all our included studies and found that only five reported such systematic methods; we did not consider diagnosis data from other studies to be robust, because they could represent suspected diagnoses rather than final diagnoses.

### Implications of this study

Apparently asymptomatic people might undergo brain and body MRI by participating in research, or access non-research MRI via referral from a doctor^28^ or directly^28^ ^32^ ^33^ (eg, as part of occupational screening,^31^ private health insurance,^23^ or company healthcare programmes^24^ ^28^). Our prevalence data could be used to inform consent for MRI in both research and non-research settings. Such data could also help researchers calculate anticipated numbers of participants with potentially serious incidental findings in future studies, to inform the design of appropriate incidental findings handling policies.

Our review highlights the limited data available on the follow-up and final diagnoses of potentially serious incidental findings. Such data would inform judgments about the benefits versus harms of feeding back potentially serious incidental findings, which warrants further investigation with systematic, long term follow-up of participants with these findings. Unlike public health screening programmes, which fulfill specific criteria to ensure net benefit,^58^ identification of a potentially serious incidental finding does not always lead to detection of disease at a stage where intervention will confer benefit. Many potentially serious incidental findings will turn out to be clinically non-serious, but require potentially anxiety provoking follow-up and potentially uncomfortable or harmful investigations to discover this. Even for those potentially serious incidental findings that do turn out to be clinically serious, for most there is no clear evidence base to inform decisions about treatment, and early treatment of some disorders might confer harm.^59^ Our prevalence data could inform power calculations for future clinical trials of conservative or active treatments of potentially serious incidental findings, in order to develop good medical practices that minimise harm to people with potentially serious incidental findings, and ensure appropriate use of health services.

**What is already known on this topic**

Estimates of prevalence of incidental findings vary widely, and could be of limited value to practice because they often include non-serious incidental findings
Previous systematic reviews have focused on incidental findings detected on magnetic resonance imaging (MRI) of a single body region, patient populations undergoing MRI, or apparently asymptomatic people imaged using another modality
These estimates are not generalisable to brain and body MRI of apparently asymptomatic people (imaging that is increasingly conducted in large scale imaging research and screening settings)

**What this study adds**

In meta-analyses of published studies, pooled prevalence of potentially serious incidental findings on MRI of apparently asymptomatic people was 3.9% for brain and body (1.4% brain, 1.3% thorax, and 1.9% abdomen), and 12.8% (1.7%, 3.0%, 4.5%, respectively) when including incidental findings of uncertain potential seriousness
Around half of potentially serious incidental findings were suspected malignancies
Limited follow-up data suggest that most potentially serious incidental findings might not be clinically serious on follow-up, and further research is needed

## Web extra

Extra material supplied by authors

**Web appendix:** Supplement

## Notes

Contributors: LMG designed and conducted the study; collected, managed, analysed, and interpreted data; and prepared, reviewed, and approved the manuscript. LP collected data and reviewed and approved the manuscript. MM advised on methods, interpreted data, and reviewed and approved the manuscript. FMC analysed and interpreted data; and prepared, reviewed, and approved the manuscript. WNW and RAS interpreted data, and reviewed and approved the manuscript. JMW designed and supervised the study, interpreted data, and reviewed and approved the manuscript. CLMS designed and supervised the study; interpreted data; reviewed, approved, and decided to submit the manuscript for publication; and is the guarantor. All authors had full access to all of the data (including statistical reports and tables) in the study and can take responsibility for the integrity of the data and the accuracy of the data analysis. The corresponding author attests that all listed authors meet authorship criteria and that no others meeting the criteria have been omitted.

Ethical approval: All data for this study were obtained from existing publications, and so did not need ethical approval.

Data sharing: The full dataset is available at http://dx.doi.org/10.7488/ds/2100 with open access.

The lead author affirms that the manuscript is an honest, accurate, and transparent account of the study being reported; that no important aspects of the study have been omitted; and that any discrepancies from the study as planned (and, if relevant, registered) have been explained.
