# Supplementary material for: Potentially serious incidental findings on brain and body magnetic resonance imaging of apparently asymptomatic adults: systematic review and meta-analysis
Source: BMJ. 2018 Nov 22;363:k4577. doi: 10.1136/bmj.k4577 (PMC6249611; doi:10.1136/bmj.k4577)
Supplement: Supplementary file 1 — Web appendix: Supplement [file gibl043550.ww.pdf]

Supplement

Table of Contents

Supplementary Methods ... 3

Supplementary Methods 1: Search strategies ... 3

Supplementary Methods 2: List used to classify incidental findings (IFs) as potentially serious or non-serious ... 5

Supplementary Methods 3: Subclassification of potentially serious incidental findings (PSIFs) ... 8

Supplementary Figures ... 9

Supplementary Figure 1: Selection of included studies ... 9

Supplementary Figure 2: Forest plots of the per-study prevalence and pooled prevalence estimates of suspected malignant incidental findings (IFs), and of suspected malignant IFs plus possible indicators of malignancy, detected on brain, thoracic, abdominal and brain and body magnetic resonance imaging (MRI) ... 11

Supplementary Figures 3a-i: Forest plots showing study-specific estimates of prevalence of potentially serious incidental findings (PSIFs) by imaged body region. Studies are ordered according to the characteristics for which we conducted subgroup analyses (i.e. imaging setting, blinding of image readers to participants' characteristics, and number of image readers) ... 15

Supplementary Figure 3a: Imaging setting – brain ... 16

Supplementary Figure 3b: Imaging setting – thorax ... 17

Supplementary Figure 3c: Imaging setting – abdomen ... 18

Supplementary Figure 3d: Imaging setting – brain and body ... 19

Supplementary Figure 3e: Blinding of readers to participants' characteristics – brain ... 20

Supplementary Figure 3f: Blinding of readers to participants' characteristics – thorax ... 21

Supplementary Figure 3g: Blinding of readers to participants' characteristics – abdomen ... 22

Supplementary Figure 3h: Blinding of readers to participants' characteristics – brain and body ... 23

Supplementary Figure 3i: Number of image readers – brain ... 24

Supplementary Tables ... 25

Supplementary Table 1: Details of included studies ordered by region imaged and descending sample size ... 26

Supplementary Table 2: Sequences evaluated for incidental findings in included studies, ordered by region imaged and descending sample size ... 28

Supplementary Table 3: 95% prediction intervals (and 95% confidence intervals to enable direct comparison) ... 31

Supplementary Tables 4a-c: Types of potentially serious incidental findings (PSIFs) by region, in descending order of frequency, as percentages of total PSIFs 32

Supplementary Table 4a: Brain 32

Supplementary Table 4b: Thorax 33

Supplementary Table 4c: Abdomen 34

Supplementary Table 5: Difference in estimates of prevalence of PSIFs between subgroups 35

Supplementary Tables 6a-c: Summary of available data on potential determinants of prevalence of potentially serious incidental findings (PSIFs, as per our definition) or IFs which required follow-up (as per each study's definition), ordered by descending sample size 36

Supplementary Table 6a: Age 37

Supplementary Table 6b: Sex 39

Supplementary Table 6c: Other factors 40

Supplementary Methods

Supplementary Methods 1: Search strategies

Search strategy 1: Incidental findings on cardiac and abdominal MRI

Databases: Embase

Ovid MEDLINE

Line number	Search term
1	exp Magnetic Resonance Imaging/
2	exp Nuclear Magnetic Resonance Imaging/
3	(magnetic resonance or MRI or MR or NMR).tw.
4	1 or 2 or 3
5	(abdom$ or cardiac or heart or cardio$ or whole-body or (whole adj2 body)).tw.
6	*Abdomen/
7	*Heart/
8	5 or 6 or 7
9	4 and 8
10	Incidental findings/
11	(incidental$ or subclinical or serendipit$ or unexpected or asymptomatic).tw.
12	10 or 11
13	Humans/
14	9 and 12 and 13
15	(Conference abstract or Conference report or Conference paper or Conference review or Case reports or comment or editorial or letter or news).pt.
16	14 not 15

Search strategy 2: Update for review of incidental findings on brain MRI

Databases: Embase

Ovid MEDLINE

Source: Adapted from Morris et al. 2009[8]

Line number	Search term
1	(MR or MRI or magnetic resonance imaging or neuroimaging).tw.
2	(screen$ or incidental$ or healthy or asymptomatic or volunteer or control).tw.
3	(cranial or brain$ or neuro$).tw.
4	*Magnetic Resonance Imaging/
5	*Brain/ or exp Brain Diseases/
6	Humans/
7	1 and 2 and 3 and 4 and 5 and 6
8	(Conference abstract or Conference report or Conference paper or Conference review or Case reports or comment or editorial or letter or news).pt.
9	7 not 8
10	limit 9 to yr="2008 -Current"
11	remove duplicates from 10

Search strategy 1 and 2 were designed/adapted respectively by Dr Lorna Gibson (MBChB) and Professor Cathie Sudlow (DPhil).

Supplementary Methods 2: List used to classify incidental findings (IFs) as potentially serious or non-serious

UK Biobank developed lists of findings which would be considered potentially serious, and findings not considered serious for use by radiographers and reporting radiologists. These lists were based on lists generated by the German National Cohort,[14] and are subject to ongoing review. These lists were used to classify incidental findings (IFs) reported in included studies as potentially serious, or non-serious.

Incidental findings on brain magnetic resonance imaging (MRI)

Potentially serious	Not serious
Acute brain infarction	Asymmetrical ventricles
Acute hydrocephalus	Chiari malformation[d]
Acute intracranial haemorrhage[a]	Chronic hydrocephalus
Arachnoid cyst[b]	Developmental anomalies (including venous anomalies)
Colloid cyst of third ventricle	Lipoma of corpus callosum
Intracranial mass lesion[c]	Non-acute brain infarction
Mastoiditis	Non-specific white matter hyperintensities
Suspected intracranial aneurysm or vascular malformation	Regional or global atrophy
	Suspected demyelination

a. Not old bleeds, or microbleeds only detected on gradient recalled echo sequences.
b. Only if large and considered likely to increase the risk of developing a subdural haematoma.
c. Except meningiomata in locations considered highly unlikely to cause problems.
d. Descent of part of the cerebellum +/- brainstem below the foramen magnum.

Incidental findings on thoracic MRI

Potentially serious	Not serious
Aortic dissection	Atelectasis
Cardiac mass (including thrombus)	Calcified pleural plaque
Central pulmonary embolus	Calcified pulmonary nodule
Haemodynamically relevant pericardial effusion > 2 cm	Emphysema
Heart valve defects[a]	Right sided descending aorta
Hilar, mediastinal, axillary or cervical lymphadenopathy[b]	
Lobar pneumonia or lung consolidation	
Lung mass > 2 cm	
Mediastinal mass > 2 cm	
Pleural effusion	
Pleural mass > 2 cm	
Pneumothorax	
Severe left or right ventricular dilation or dysfunction	
Severe left ventricular hypertrophy > 2 cm thick wall	
Thoracic aortic aneurysm > 5 cm	

a. Severe regurgitation jet of any valve or severe turbulence (suggesting valve stenosis).
b. > 1.5 cm and > 3 lymph nodes grouped in a circumscribed region.

Incidental findings on abdominal MRI

**Potentially serious**	**Not serious**
Abdominal aortic aneurysm > 5 cm	Abdominal wall hernia
Acute exudative pancreatitis	Bladder diverticulum
Adrenal lesion > 2 cm	Chronic cholecystitis
Ascites	Chronic pancreatitis
Cholestasis (intra- or extra-hepatic)[a]	Fatty liver
Deep vein thrombosis	Fibroids
Hepatomegaly	Gallstones
Ileus	Hiatus hernia
Intra-abdominal mass > 3 cm	Left sided inferior vena cava
Irregular/nodular liver margin	Liver cyst
Lymphadenopathy[b]	Renal calculus
Multiple small non-cystic, liver lesions (non haemangioma-like)	Simple renal cyst
Pneumoperitoneum	Single kidney
Portal vein occlusion	
Pyelonephritis	
Renal artery stenosis > 80% or bilateral	
Solid/cystic pancreatic tumour	
Solid gallbladder lesion	
Solid liver lesion	
Solid/semi-solid renal tumour > 2 cm	
Spleen infarction	
Splenomegaly > 15 cm	
Urinary obstruction	
Urinary tract mass > 2 cm	

a. Common bile duct > 15 mm (or > 20 mm post-cholecystectomy).
b. > 1.5 cm and > 3 lymph nodes grouped in a circumscribed region.

Supplementary Methods 3: Subclassification of potentially serious incidental findings (PSIFs)

Suspected malignancy

We subclassified PSIFs as suspected malignancy if they described tumours, masses, complex cysts or lesions.

Possible indicator of malignancy

Other PSIFs which may be indicative of malignancy are those which are not masses, and may be related to either malignancy, or another aetiology. These included pleural and pericardial effusions, enlarged lymph nodes, hydronephrosis, splenomegaly, biliary dilatation, ascites, hydrocephalus and severe bone oedema.

Non-malignant

If a PSIF could not be classified as either suspected malignancy, or as a possible indicator of malignancy, we subclassified it as non-malignant.

Supplementary Figures

Supplementary Figure 1: Selection of included studies

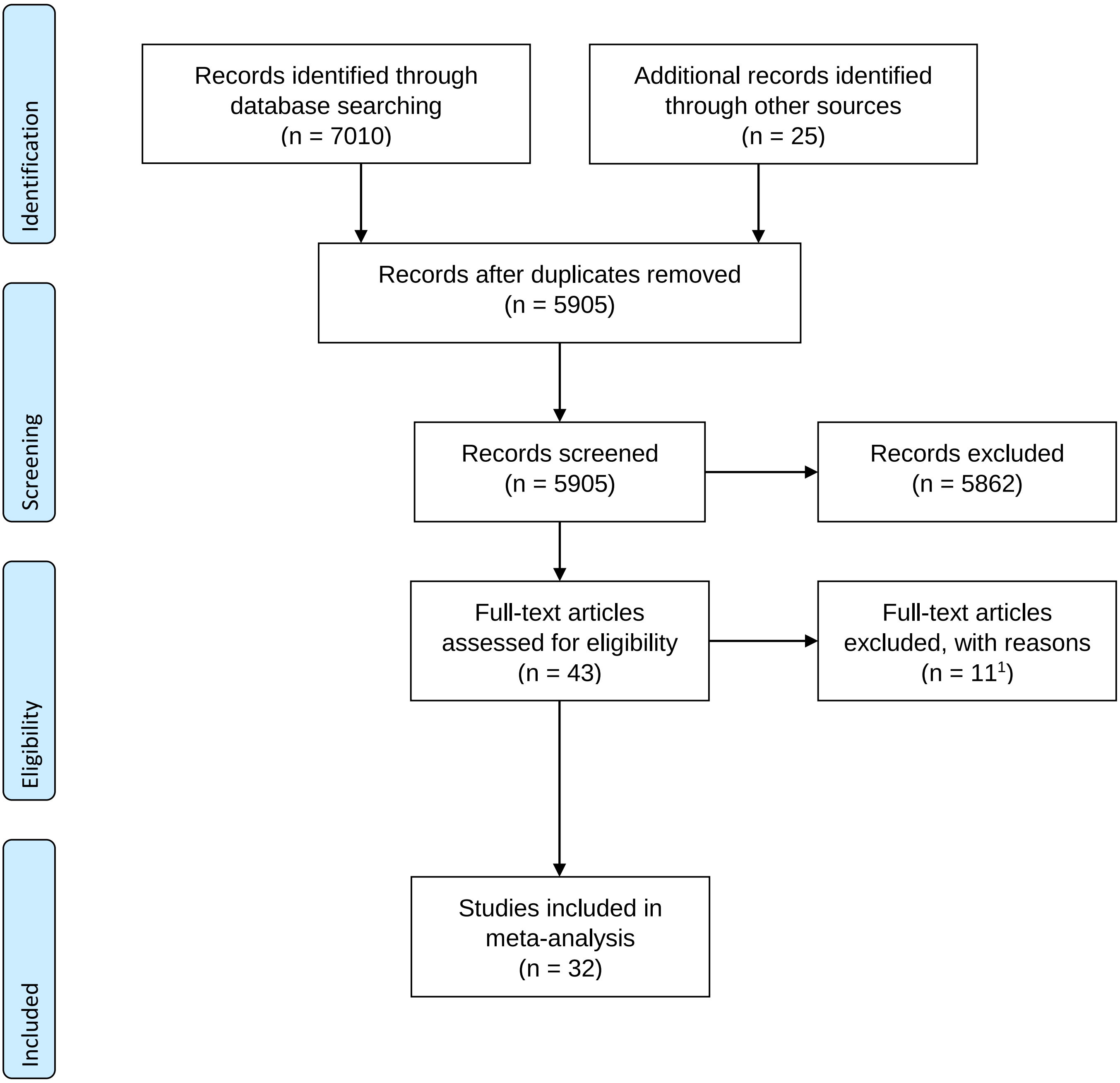

1. Eleven studies were excluded after reviewing the full-text article: two were superseded by larger cohorts reported in other articles; two included patients and apparently asymptomatic volunteers and did not report data separately for apparently asymptomatic volunteers; two did not report the age of participants; two included adults and children and did not report data separately for adults; one was a study of children; one investigated a single type of incidental finding; one did not report the age of participants undergoing brain imaging, and it was not clear if participants were apparently asymptomatic volunteers or not.

Supplementary Figure 2: Forest plots of the per-study prevalence and pooled prevalence estimates of suspected malignant incidental findings (IFs), and of suspected malignant IFs plus possible indicators of malignancy, detected on brain, thoracic, abdominal and brain and body magnetic resonance imaging (MRI)

Region imaged and study name	Sample size	Suspected malignant IFs[a,c]		Suspected malignant IFs and possible indicators of malignancy[b,c]	
		n	Prevalence, % (95% CI)	n	Prevalence, % (95% CI)
**Brain**					
Bos 2016[29]	5800	183	3.2 (2.7 to 3.6)		
Yue 1997[30]	3672	8	0.2 (0.1 to 0.4)		
Weber 2006[31]	2536	9	0.4 (0.2 to 0.7)		
Hegenscheid 2013[21]	2500	26	1.0 (0.7 to 1.5)		
Lee 2008[32]	2164	2	0.1 (0.0 to 0.3)		
Tsushima 2005[33]	1113	4	0.4 (0.1 to 0.9)		
Baumgart 2007[22]	1007	11	1.1 (0.5 to 1.9)		
Haberg 2016[34]	1006	5	0.5 (0.2 to 1.2)		
Katzman 1999[35]	1000	3	0.3 (0.1 to 0.9)		
Sommer 2013[36]	722	4	0.6 (0.2 to 1.4)		
Sandeman 2013[37]	700	6	0.9 (0.3 to 1.9)		
Cieszanowski 2014[23]	666	0	0.0 (0.0 to 0.6)		
Alphs 2006[38]	656	14	2.1 (1.2 to 3.6)	19	2.9 (1.8 to 4.5)
Brugulat-Serrat 2017[39]	575	6	1.0 (0.4 to 2.3)		
Hoggard 2009[40]	525	5	1.0 (0.3 to 2.2)		
Boutet 2016[41]	503	13	2.6 (1.4 to 4.4)		
Kumar 2008[42]	478	5	1.0 (0.3 to 2.4)		
Goehde 2005[24]	298	2	0.7 (0.1 to 2.4)		
Hartwigsen 2010[43]	206	8	3.9 (1.7 to 7.5)		
Reneman 2012[44]	203	1	0.5 (0.0 to 2.7)		
Li 2015[45]	167	0	0.0 (0.0 to 2.2)		
Illes 2004[46]	151	0	0.0 (0.0 to 2.4)		
Morin 2009[25]	148	0	0.0 (0.0 to 2.5)		
Lo 2008[26]	132	0	0.0 (0.0 to 2.8)		
Wahlund 1989[47]	101	0	0.0 (0.0 to 3.6)		
Menzler 2010[48]	100	1	1.0 (0.0 to 5.4)		
Lubman 2002[49]	98	1	1.0 (0.0 to 5.6)		
Trufyn 2014[50]	56	0	0.0 (0.0 to 6.4)		
Saya 2017[27]	44	0	0.0 (0.0 to 8.0)		
Tarnoki 2015[28]	22	0	0.0 (0.0 to 15.4)		
**Combined**	**27349**	**317**	**0.6 (0.4 to 0.9)**	**322**	**0.6 (0.4 to 0.9)**
Heterogeneity			*T^2 = 0.9*		*T^2 = 0.9*

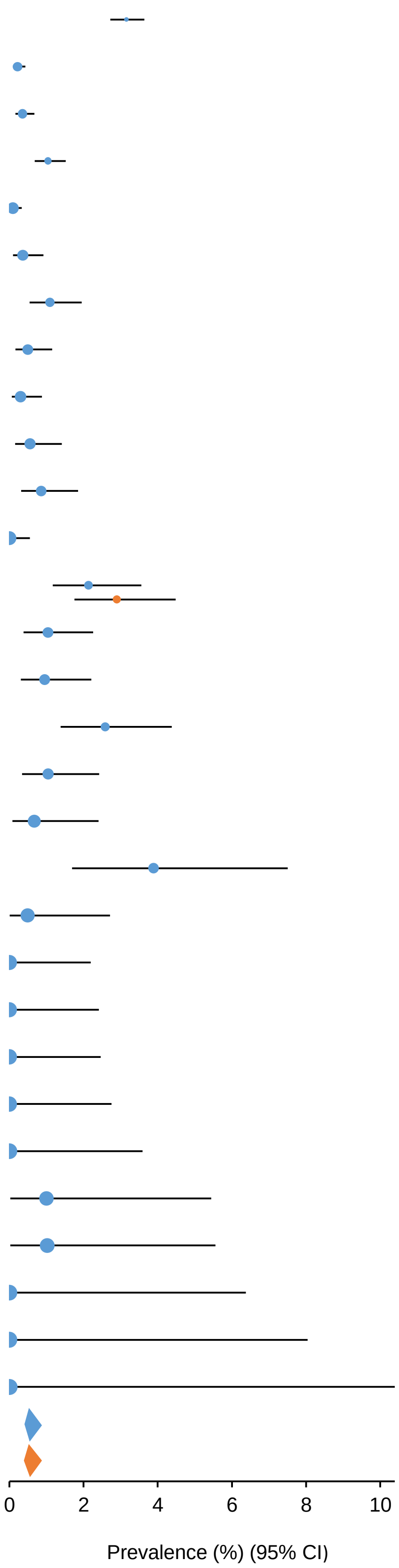

Region imaged and study name	Sample size	Suspected malignant IFs[a,c] n	Suspected malignant IFs[a,c] Prevalence, % (95% CI)	Suspected malignant IFs and possible indicators of malignancy[b,c] n	Suspected malignant IFs and possible indicators of malignancy[b,c] Prevalence, % (95% CI)
**Thorax**					
Hegenscheid 2013[21]	2500	114	4.6 (3.8 to 5.5)	138	5.5 (4.7 to 6.5)
Baumgart 2007[22]	1007	1	0.1 (0.0 to 0.6)		
Cieszanowski 2014[23]	666	0	0.0 (0.0 to 0.6)		
Goehde 2005[24]	298	5	1.7 (0.5 to 3.9)		
Vogel-Claussen 2009[51]	254	6	2.4 (0.9 to 5.1)	19	7.5 (4.6 to 11.4)
Morin 2009[25]	148	0	0.0 (0.0 to 2.5)	1	0.7 (0.0 to 3.7)
Lo 2008[26]	132	5	3.8 (1.2 to 8.6)		
Saya 2017[27]	44	0	0.0 (0.0 to 8.0)		
Loy 2015[52]	40	0	0.0 (0.0 to 8.8)		
Tarnoki 2015[28]	22	1	4.5 (0.1 to 22.8)	4	18.2 (5.2 to 40.3)
**Combined**	**5111**	**132**	**0.6 (0.1 to 3.1)**	**173**	**1.0 (0.2 to 5.4)**
Heterogeneity			*T^2 = 2.8*		*T^2 = 3.9*
**Abdomen**					
Hegenscheid 2013[21]	2500	302	12.1 (10.8 to 13.4)	354	14.2 (12.8 to 15.6)
Baumgart 2007[22]	1007	3	0.3 (0.1 to 0.9)		
Cieszanowski 2014[23]	666	0	0.0 (0.0 to 0.6)		
Goehde 2005[24]	298	7	2.3 (0.9 to 4.8)	8	2.7 (1.2 to 5.2)
Morin 2009[25]	148	1	0.7 (0.0 to 3.7)	2	1.4 (0.2 to 4.8)
Lo 2008[26]	132	7	5.3 (2.2 to 10.6)		
Saya 2017[27]	44	0	0.0 (0.0 to 8.0)		
Tarnoki 2015[28]	22	5	22.7 (7.8 to 45.4)	6	27.3 (10.7 to 50.2)
**Combined**	**4817**	**325**	**1.3 (0.2 to 9.3)**	**380**	**1.6 (0.2 to 10.9)**
Heterogeneity			*T^2 = 4.4*		*T^2 = 4.5*
**Brain and body**					
Hegenscheid 2013[21]	2500	459	18.4 (16.9 to 21.9)	558	22.3 (20.7 to 24.0)
Baumgart 2007[22]	1007	15	1.5 (0.8 to 2.4)		
Cieszanowski 2014[23]	666	0	0 (0 to 0.6)		
Goehde 2005[24]	298	14	4.7 (2.6 to 7.8)	15	5.0 (2.8 to 8.2)
Morin 2009[25]	148	1	0.7 (0.0 to 3.7)	3	2.0 (0.4 to 5.8)
Lo 2008[26]	132	14	10.6 (5.9 to 17.2)		
Saya 2017[27]	44	0	0.0 (0.0 to 8.0)		
Tarnoki 2015[28]	22	6	27.3 (10.7 to 50.2)	10	45.5 (24.4 to 67.8)
**Combined**	**4817**	**509**	**2.3 (0.3 to 15.4)**	**615**	**3.0 (0.4 to 20.4)**
Heterogeneity			*T^2 = 4.5*		*T^2 = 5.0*

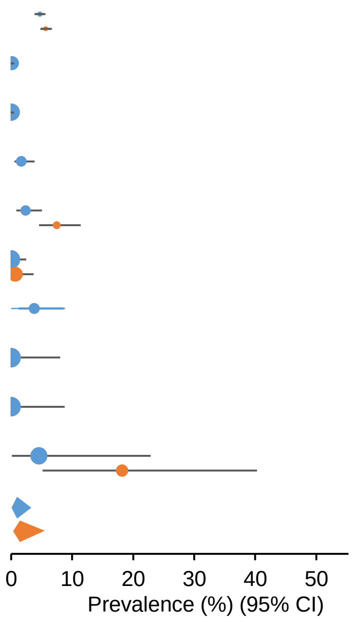

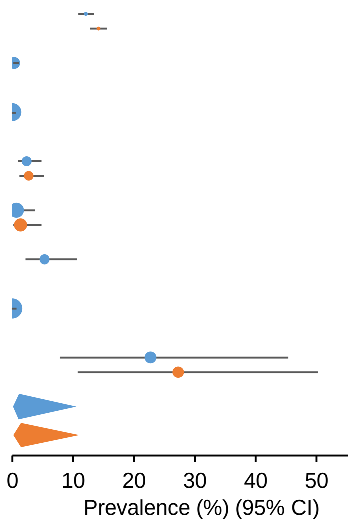

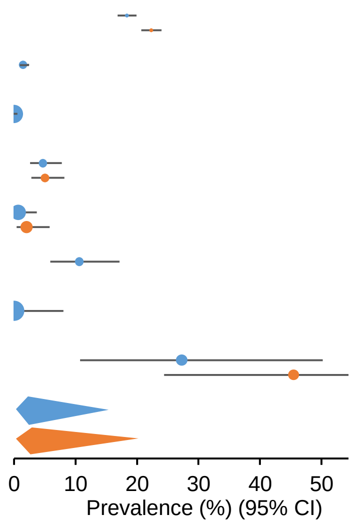

IFs = incidental findings, CI= confidence interval

Tau-squared is an estimate of between-study variance on the logit scale. Zero represents no variance, and increasing values of tau-squared indicate increasing heterogeneity.

Blue = Per-study point prevalence and pooled prevalence estimate of suspected malignant IFs on brain, thoracic, abdominal and brain and body magnetic resonance imaging (MRI)

Orange = Sensitivity analyses which include IFs classed as possible indicators of malignancy in the per-study point prevalence and pooled prevalence estimate of suspected malignant IFs on brain, thoracic, abdominal and brain and body MRI

a. Suspected malignant IFs include tumours, masses, complex cysts and lesions
b. Possible indicators of malignancy include pleural and pericardial effusions, enlarged lymph nodes, hydronephrosis, splenomegaly, biliary dilatation, ascites, hydrocephalus and severe bone oedema
c. As per Figure 1, we excluded IFs detected in studies that used specialist imaging sequences (97 breast lesions in a study including MR mammography,[21] and 87 colonic polyps in two studies which included MR colonography[22 24]) from pooled analyses.

Supplementary Figures 3a-i: Forest plots showing study-specific estimates of prevalence of potentially serious incidental findings (PSIFs) by imaged body region. Studies are ordered according to the characteristics for which we conducted subgroup analyses (i.e. imaging setting, blinding of image readers to participants' characteristics, and number of image readers)

Supplementary Figure 3a: Imaging setting – brain

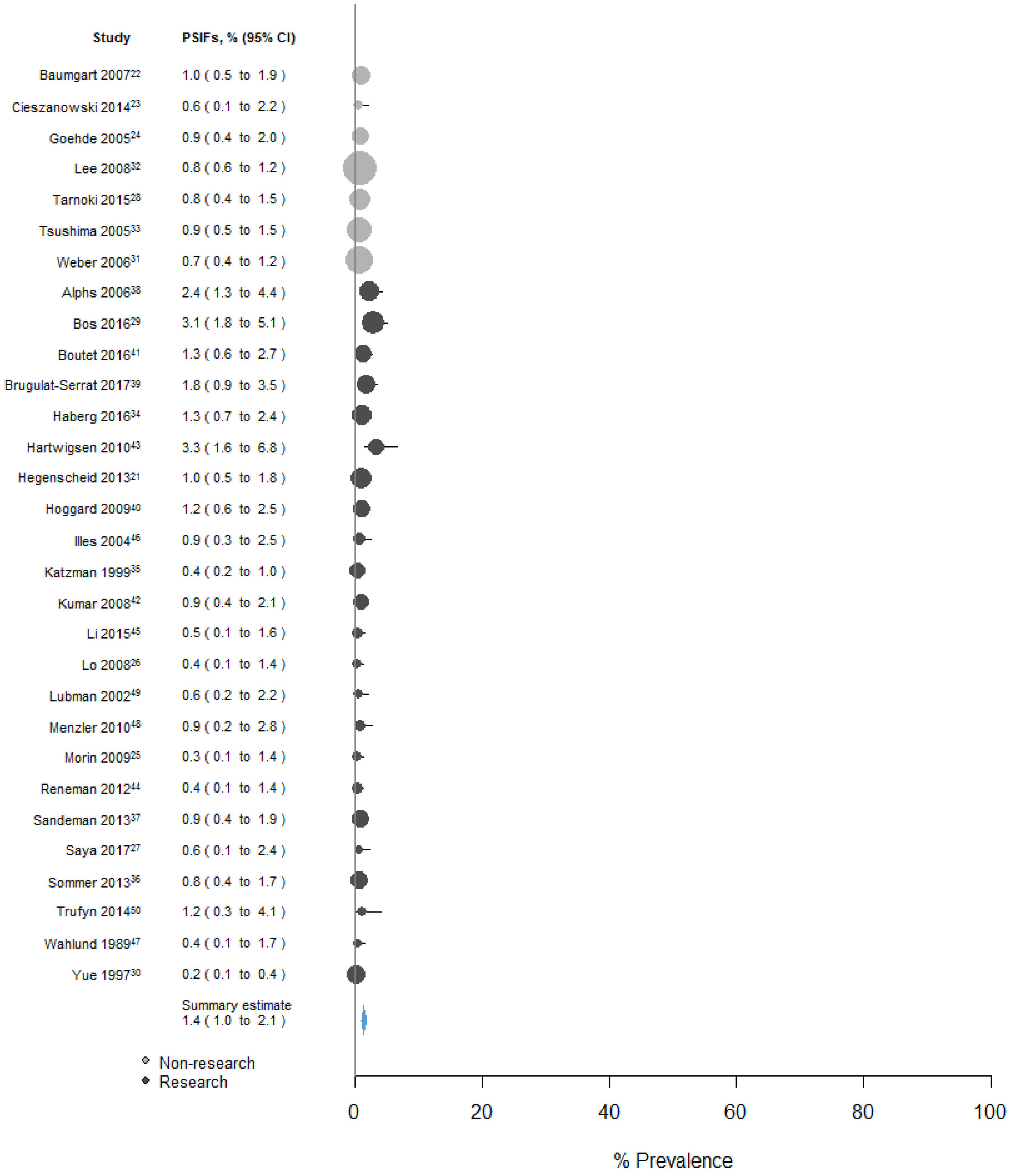

PSIFs = potentially serious incidental findings, CI = confidence interval

We defined research imaging as that performed as part of a research study. We classified other imaging of apparently asymptomatic participants as non-research (i.e. studies of occupational screening, commercial screening [i.e. paid for by the participant] or medical screening [whether referred by a doctor, or self-referred, or provided by health insurance]).

Supplementary Figure 3b: Imaging setting – thorax

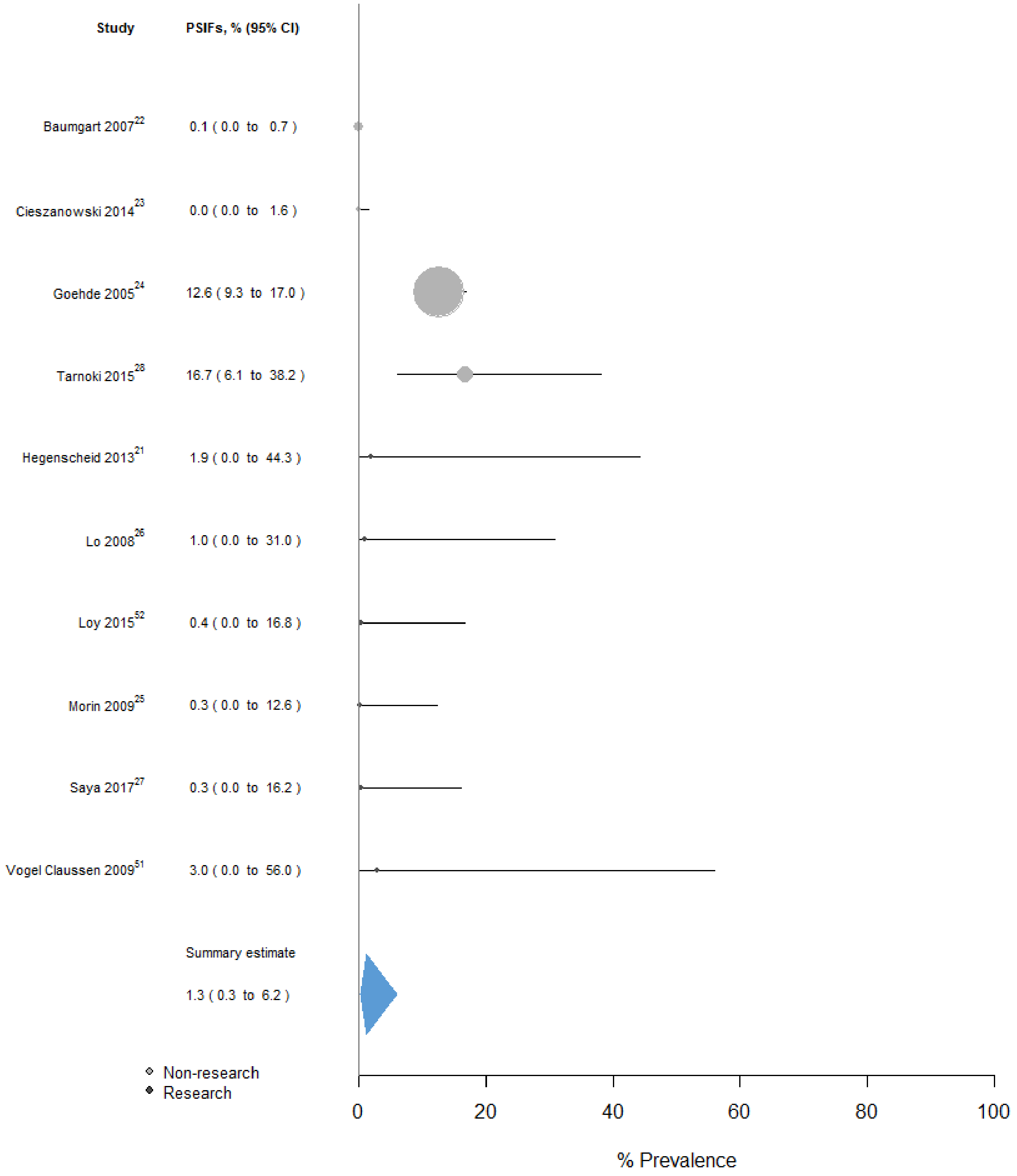

PSIFs = potentially serious incidental findings, CI = confidence interval

We defined research imaging as that performed as part of a research study. We classified other imaging of apparently asymptomatic participants as non-research (i.e. studies of occupational screening, commercial screening [i.e. paid for by the participant] or medical screening [whether referred by a doctor, or self-referred, or provided by health insurance]).

Supplementary Figure 3c: Imaging setting – abdomen

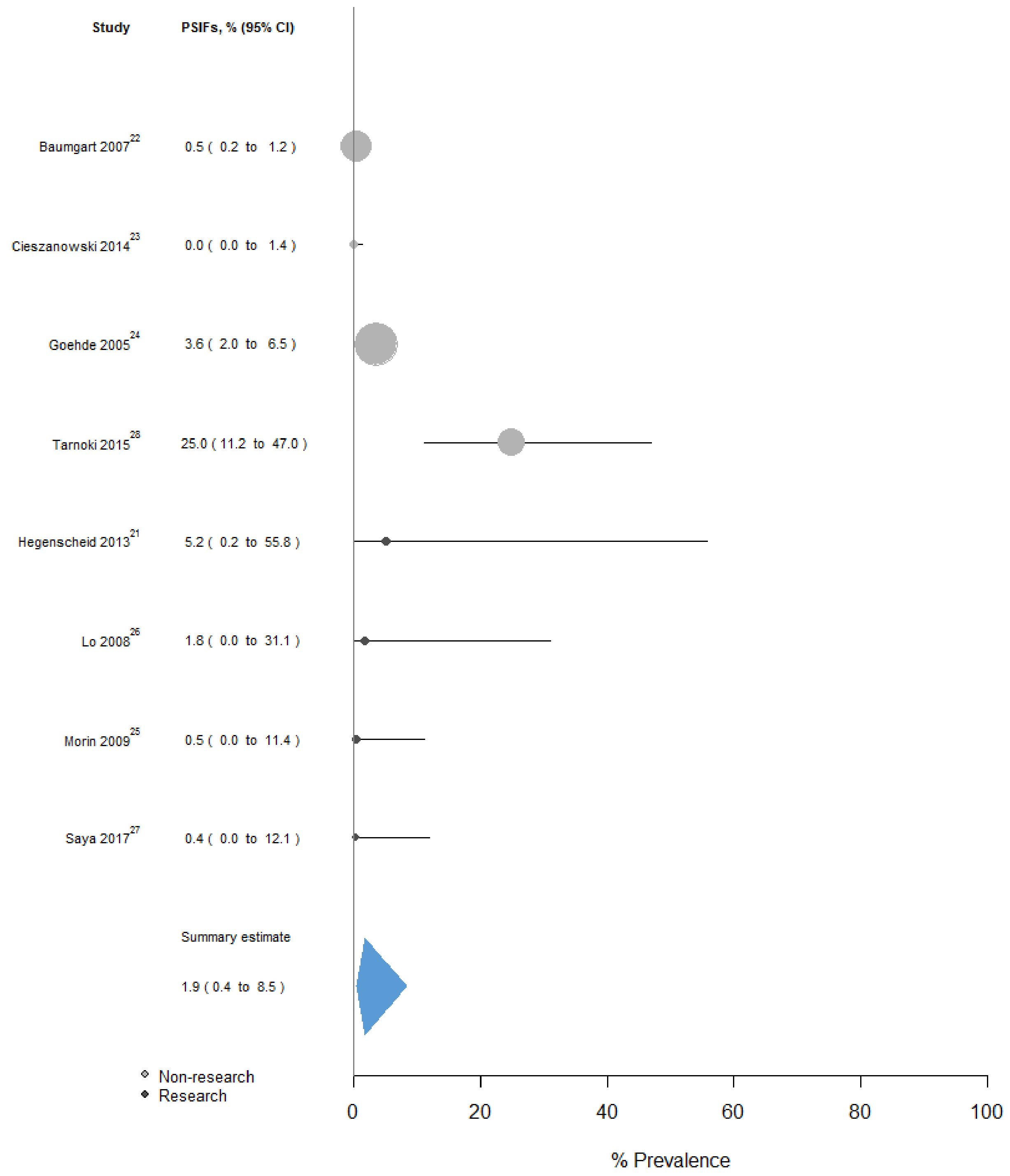

PSIFs = potentially serious incidental findings, CI = confidence interval

We defined research imaging as that performed as part of a research study. We classified other imaging of apparently asymptomatic participants as non-research (i.e. studies of occupational screening, commercial screening [i.e. paid for by the participant] or medical screening [whether referred by a doctor, or self-referred, or provided by health insurance]).

Supplementary Figure 3d: Imaging setting – brain and body

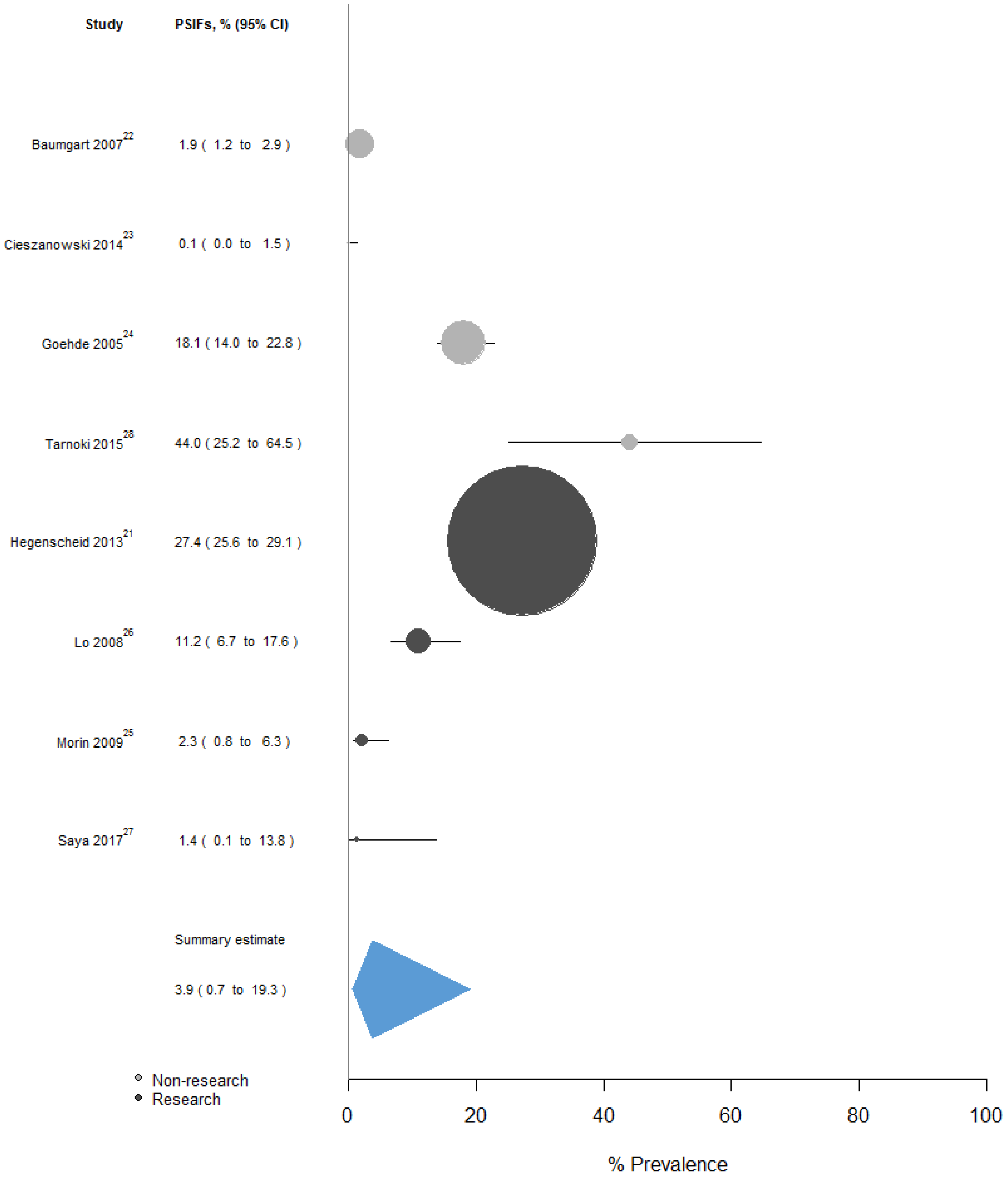

PSIFs = potentially serious incidental findings, CI = confidence interval

We defined research imaging as that performed as part of a research study. We classified other imaging of apparently asymptomatic participants as non-screening (i.e. studies of occupational screening, commercial screening [i.e. paid for by the participant] or medical screening [whether referred by a doctor, or self-referred, or provided by health insurance]).

Supplementary Figure 3e: Blinding of readers to participants' characteristics – brain

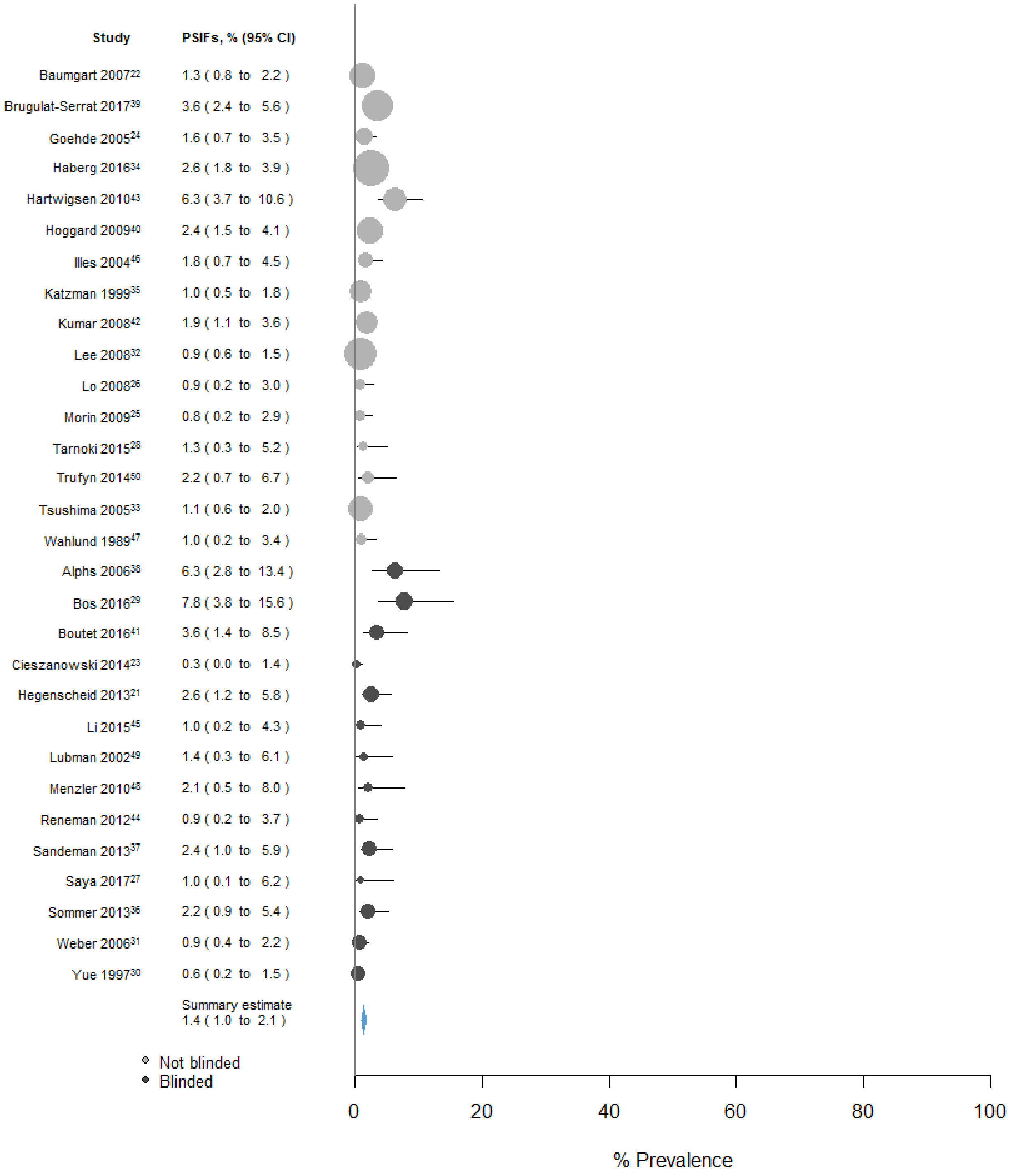

PSIFs = potentially serious incidental findings, CI = confidence interval

We classified studies which reported they did not blind readers, or studies who did not provide information on blinding, as not blinded for the purposes of these subgroup analyses.

Supplementary Figure 3f: Blinding of readers to participants' characteristics – thorax

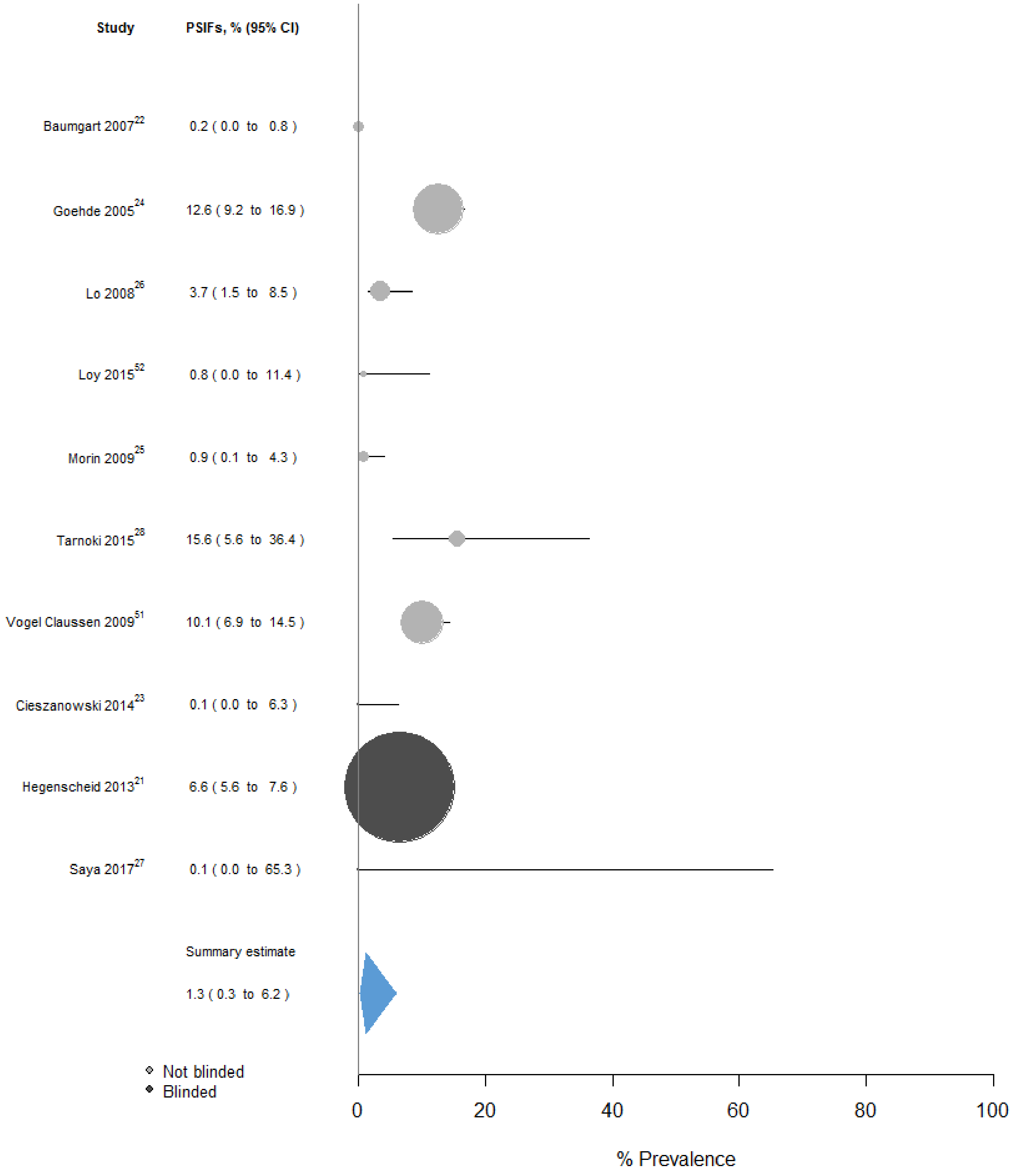

PSIFs = potentially serious incidental findings, CI = confidence interval

We classified studies which reported they did not blind readers, or studies who did not provide information on blinding, as not blinded for the purposes of these subgroup analyses.

Supplementary Figure 3g: Blinding of readers to participants' characteristics – abdomen

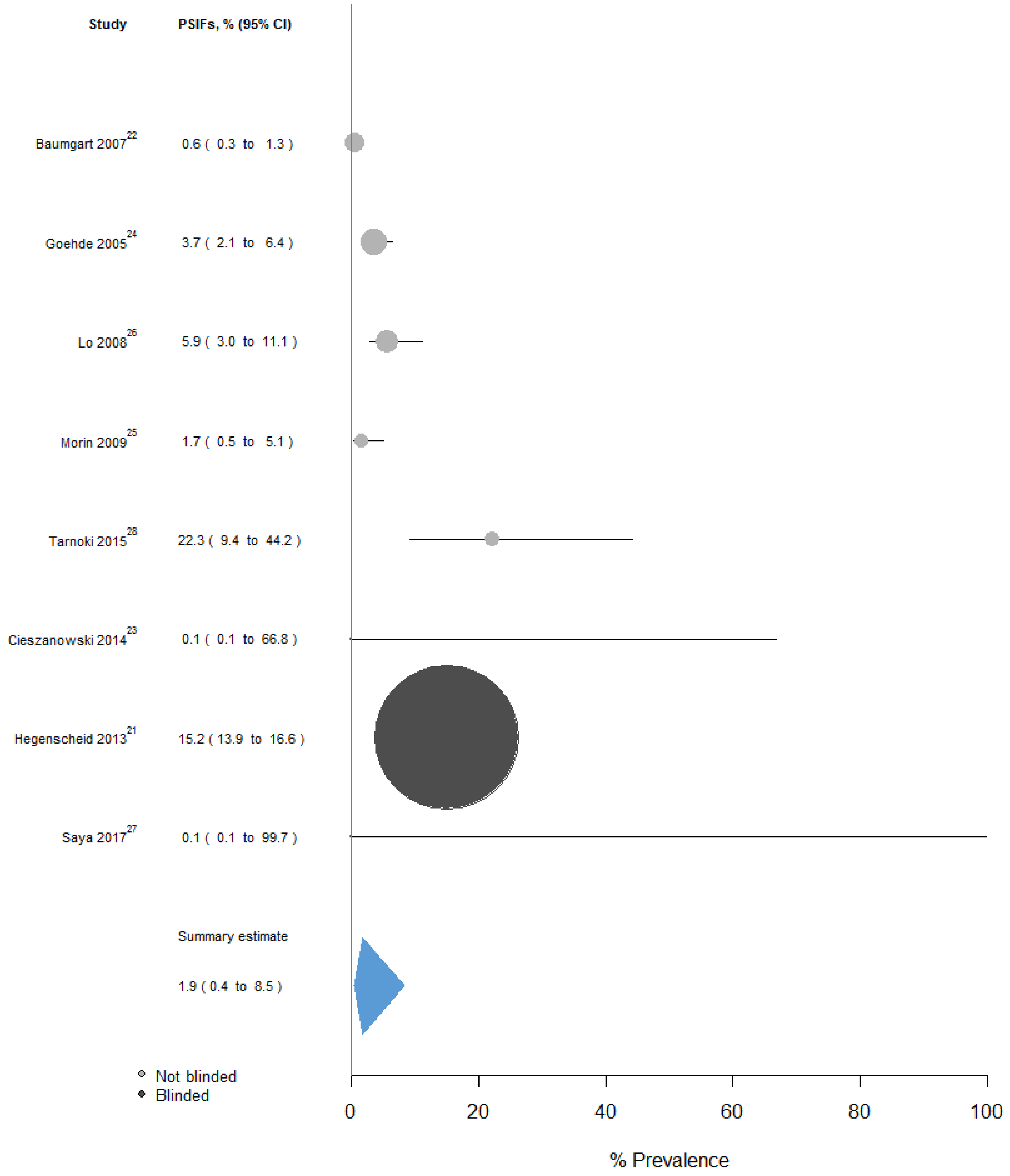

PSIFs = potentially serious incidental findings, CI = confidence interval

We classified studies which reported they did not blind readers, or studies who did not provide information on blinding, as not blinded for the purposes of these subgroup analyses.

Supplementary Figure 3h: Blinding of readers to participants' characteristics – brain and body

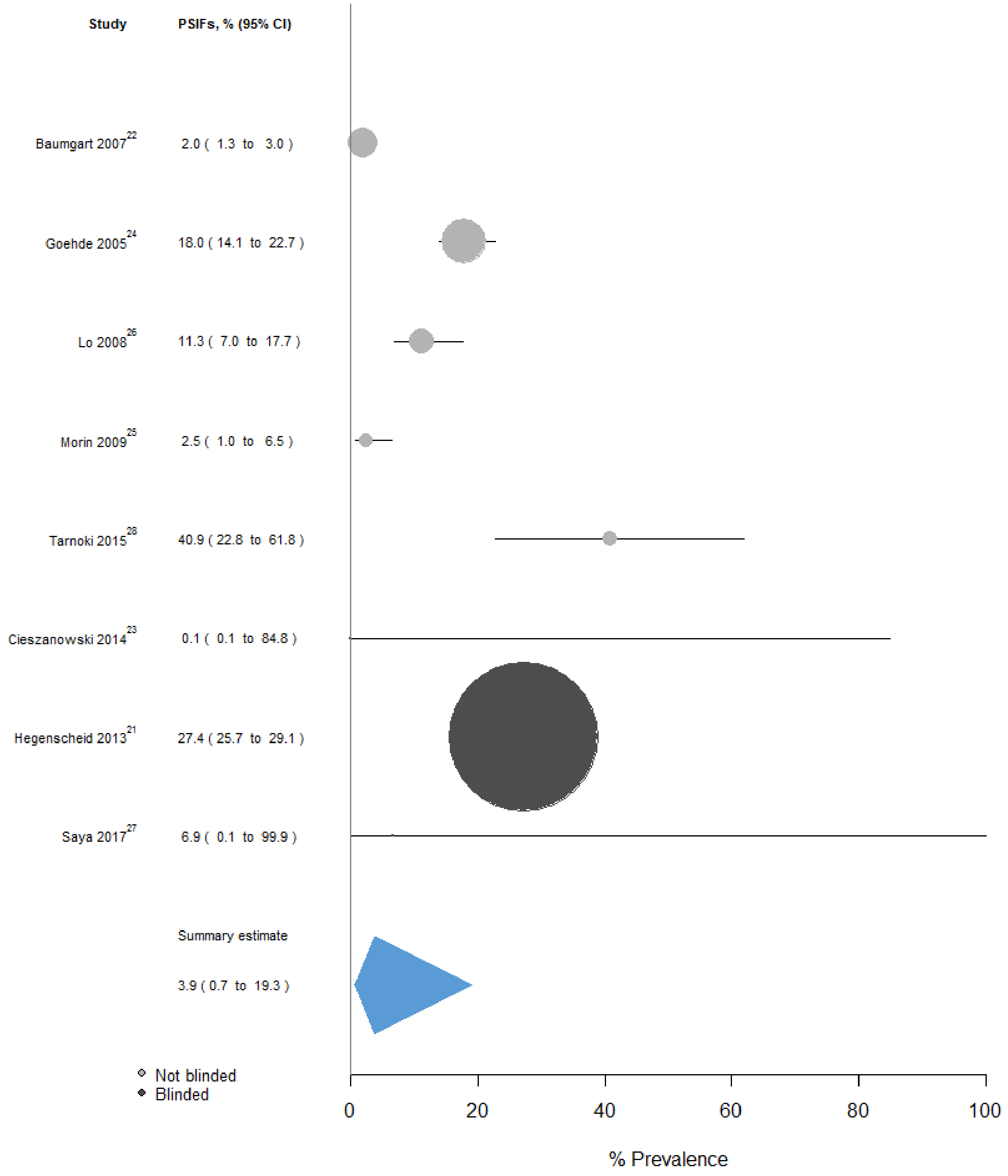

PSIFs = potentially serious incidental findings, CI = confidence interval

We classified studies which reported they did not blind readers, or studies who did not provide information on blinding, as not blinded for the purposes of these subgroup analyses.

Supplementary Figure 3i: Number of image readers – brain

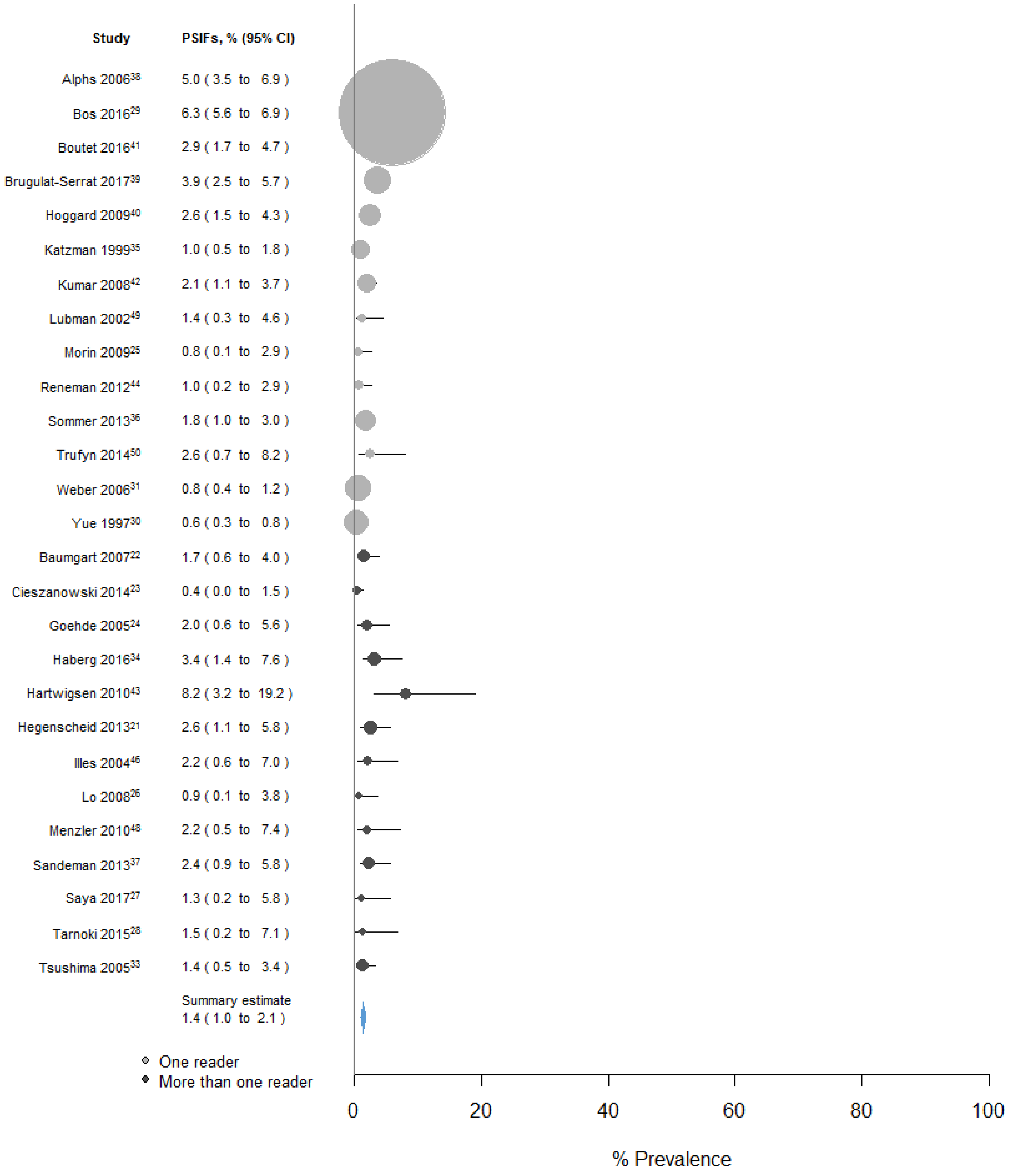

PSIFs = potentially serious incidental findings, CI = confidence interval

Number of image readers refers to the numbers of readers who assessed all images for IFs. Three studies of brain PSIFs (2432/27349 [8.9%] participants)[32,45,47] did not provide data on number of image readers and were excluded from this subgroup analysis. There were sparse data within subgroups of studies involving one image reader for thorax, abdomen, and brain and body studies (PSIFs were present in only 1/188, 2/248 and 3/148 participants respectively); therefore these subgroup analyses were not performed.

Supplementary Tables

Supplementary Table 1: Details of included studies ordered by region imaged and descending sample size

	Study population variables					Imaging variables				
								Readers		
**Study first author and publication year**	**N[a]**	**n (%) male**	**Mean age (range)**	**Random sampling[b]**	**Country**	**Imaging setting**	**Data source[c]**	**Specialities**	**N**	**Blinded[d]**
**Brain and body MRI**										
Hegenscheid 2013[21]	2500	1229 (49)	53 (21-88)	Yes	Germany	Longitudinal cohort	I	Radiologist	>1	Yes
Baumgart 2007[22]	1007	715 (71)	55 (40-67)	No	Germany	Screening	I	Radiologist	>1	-
Cieszanowski 2014[23]	666	465 (70)	46 (20-77)	No	Poland	Screening	I	Radiologist	>1	Yes
Goehde 2005[24]	298	247 (83)	50 (31-73)	No	Germany	Screening	I	Radiologist	>1	No
Morin 2009[25]	148	94 (64)	36[e] (21-69)	No	UK	Other research	I	Radiologist	1[f]	-
Lo 2008[26]	132	111 (84)	56 (38-82)	No	Hong Kong	Other research	I	Radiologist	>1	-
Saya 2017[27,g]	44	17 (39)	38[e] (22-59)	No	UK	Other research	I	Radiologist	>1	Yes
Tarnoki 2015[28]	22	18 (82)	47 (-)	No	Germany	Screening	I	Radiologist	>1	-
**Brain MRI only**										
Bos 2016[29]	5800	2606 (45)	65 (-)	-	Netherlands	Longitudinal cohort	I	Trained readers[h]	1	Yes
Yue 1997[30]	3672	1531 (42)	- (≥65)	No	USA	Longitudinal cohort	I	Neuroradiologist	1	Yes
Weber 2006[31]	2536	2536 (100)	21 (17-35)[i]	No	Germany	Screening	I	Radiologist	1	Yes
Lee 2008[32]	2164	1234 (57)	52 (17-89)[i]	No	Taiwan	Screening	-	Neuro- or general radiologist	-	-
Tsushima 2005[33]	1113	761 (68)	53 (22-84)	No	Japan	Screening	I	Radiologist and a neurosurgeon	>1	-
Haberg 2016[34]	1006	476 (47)	59[d] (51-67)	No	Norway	Longitudinal cohort	I	Neuroradiologist	>1	No
Katzman 1999[35]	1000	546 (55)	31 (3-83)[i]	No	USA	Other research	I	Neuroradiologist	1	-
Sommer 2013[36,g]	722	407 (56)	34 (-)	No	Netherlands	Other research	I	Neuroradiologist	1	Yes
Sandeman 2013[37]	700	368 (53)	73 (-)	No	UK	Longitudinal cohort	I	Neuroradiologist	>1	Yes
Alphs 2006[38]	656	656 (100)	61 (35-82)	No	USA	Longitudinal cohort	I	Neuroradiologist	1	Yes
Brugulat-Serrat 2017[39]	575	227 (40)	59 (45-75)	No	Spain	Longitudinal cohort	I	Neuroradiologist	1	-
Hoggard 2009[40]	525	330 (63)	35 (-)	No	UK	Other research	I	Neuroradiologist	1	-
Boutet 2016[41]	503	208 (41)	75 (71-79)	No	France	Longitudinal cohort	I	Neuroradiologist	1	Yes
Kumar 2008[42]	478	252 (53)	- (60-64)	-	Australia	Longitudinal cohort	I	Radiologist[j]	1[j]	-
Hartwigsen 2010[43]	206	117 (57)	26 (9-50)[i]	No	Germany	Other research	I	Neuroradiologist	>1	-
Reneman 2012[44]	203	92 (45)	22 (18-35)	No	Netherlands	Other research	I	Neuroradiologist	1	Yes
Li 2015[45,g]	167	0 (0)	24 (-)	No	Taiwan	Other research	I	MRI operators[k]	-[k]	Yes

	Study population variables					Imaging variables				
								Readers		
Study first author and publication year	N[a]	n (%) male	Mean age (range)	Random sampling[b]	Country	Reason for imaging	Data source[c]	Specialities	N	Blinded[d]
**Brain MRI only, continued**										
Illes 2004[46]	151	82 (54)	47 (18-90)	No	USA	Other research	I	Neuroradiologist	>1	-
Wahlund 1989[47g]	101	- (-)	- (16-87)[i]	-	Sweden	Other research	-	-	-	-
Menzler 2010[48]	100	42 (42)	29 (-)	-	Germany	Other research	I	Neuroradiologist	>1	Yes
Lubman 2002[49,g]	98	62 (63)	27 (-)	No	Australia	Other research	R	Neuroradiologist	1	Yes
Trufyn 2014[50,g]	56	15 (27)	45 (-)	No	Canada	Other research	I	Neuroradiologist	1	-
**Cardiac MRI only**										
Vogel Claussen 2009[51]	254	124 (49)	61 (45-89)	No	USA	Longitudinal cohort	I	Radiologist	>1	-
Loy 2015[52,g]	40	40 (100)	46 (-)	No	Ireland	Other research	I	Cardiologist	1	-

MRI = magnetic resonance imaging, I = imaging, UK = United Kingdom, USA = United States of America, R = reports, - = Information not specified or not sufficiently well described

a. Sample size indicates the number of apparently asymptomatic volunteers imaged, as some studies also included patient groups (see footnote g).
b. Indicates whether or not the participants were randomly sampled from the base population.
c. Indicates whether information on incidental findings (IFs) was determined from review of images (I), or reports (R), or not specified (-).
d. Blinded to information about participants.
e. Median age; no data on mean age were available.
f. Morin 2009:[25] All scans were reviewed by a single radiologist, and only scans with a potentially highly significant abnormality were reviewed by two radiologists.
g. Study included groups of patients and apparently asymptomatic volunteers, only data from the apparently asymptomatic volunteers were included in this review.
h. Bos 2016:[29] All scans were reviewed by a group of trained readers (researchers or neuropsychologists) for IFs. Two neuroradiologists reviewed only scans with suspected IFs. We have assumed that the reported data on IFs pertain to those confirmed by the neuroradiologists.
i. Study was included in the review, as it was deemed to involve only a small proportion of children, as judged in consensus by two authors (LG and CLMS) based on available data on the age of the study population.
j. Kumar 2008:[42] All scans were reviewed by a single radiologist, and only scans with suspected abnormalities were reviewed by a second reader, a neuropsychiatrist.
k. Li 2015:[45] All scans were reviewed by an MRI operator for IFs, and confirmed by a neuroradiologist, blinded to participants' clinical status. It is not clear if the neuroradiologist reviewed all participants' scans for IFs, or just those with abnormalities detected by the MRI operator. We have assumed that the reported data on IFs pertain to those confirmed by the neuroradiologist.

Supplementary Table 2: Sequences evaluated for incidental findings in included studies, ordered by region imaged and descending sample size

Study	Sample size N	Magnet strength (Tesla)	Single protocol used to image participants[a]	Brain MRI						Thoracic MRI				Abdominal MRI				Other[c]
				Contrast	T1	T2	FLAIR	Haem sensitive[b]	MRA	Contrast	Non-contrast	Cardiac specific	MRA	Contrast	T1	T2 or STIR	MRA	
**Brain and body MRI**																		
Hegenscheid 2013[21]	2500	1.5	Y	N	Y	Y	Y	Y	Y	Y	Y	Y	Y	N	Y	Y	Y	Y
Baumgart 2007[22]	1007	1.5	Y	Y	Y	Y	.	.	Y	Y	Y	Y	Y	.	Y	Y[d]	Y	Y
Cieszanowski 2014[23]	666	1.5	Y	.	.		Y	.	.	.	Y	.	.	.	Y	Y	.	Y
Goehde 2005[24]	298	1.5	Y	.	Y	Y	Y	.	Y	Y	Y	Y	Y	.	Y	.	Y	Y
Morin 2009[25]	148	1.5	Y	.	Y	.	.	.	.	.	Y	.	.	.	Y	Y	.	.
Lo 2008[26]	132	3.0	Y	N	Y	Y	.	.	.	N	Y	.	.	N	Y	Y	.	Y
Saya 2017[27]	44	1.5	Y	N	Y	Y	.	.	.	N	Y	.	.	N	Y	Y	.	Y
Tarnoki 2015[28]	22	3.0	Y	.	Y	.	.	.	Y	.	Y	.	Y	.	Y	Y	Y	Y
**Brain MRI only**																		
Bos 2016[29]	5800	1.5	Y	N	Y	.	Y	Y	.	.	.	.	.	.	.	.	.	Y
Yue 1997[30]	3672	0.35/1.5[e]	Y	N	Y	Y	.	.	.	.	.	.	.	.	.	.	.	Y
Weber 2006[31]	2536	1.0	Y	Y[d]	Y	Y	Y	.	Y[d]	.	.	.	.	.	.	.	.	.
Lee 2008[32]	2164	1.5	Y	.	Y	Y	Y	.	Y[d]	.	.	.	.	.	.	.	.	.
Tsushima 2005[33]	1113	1.0	Y	N	Y	Y	.	.	Y	.	.	.	.	.	.	.	.	Y
Haberg 2016[34]	1006	1.5	Y	.	Y	Y	Y	Y	Y	.	.	.	.	.	.	.	.	.
Katzman 1999[35]	1000	NS	N[f]	.	Y	Y	.	.	.	.	.	.	.	.	.	.	.	Y

Study (continued from above)	Sample size N	Magnet strength (Tesla)	Single protocol used to image participants[a]	Brain MRI: Contrast	Brain MRI: T1	Brain MRI: T2	Brain MRI: FLAIR	Brain MRI: Haem sensitive[b]	Brain MRI: MRA	Thoracic MRI: Contrast	Thoracic MRI: Non-contrast	Thoracic MRI: Cardiac specific	Thoracic MRI: MRA	Abdominal MRI: Contrast	Abdominal MRI: T1	Abdominal MRI: T2 or STIR	Abdominal MRI: MRA	Other[c]
Sommer 2013[36]	722	1.5	N[g]	.	.	Y	.	.	.	.	.	.	.	.	.	.	.	.
Sandeman 2013[37]	700	1.5	Y	.	Y	Y	Y	Y	.	.	.	.	.	.	.	.	.	.
Alphs 2006[38]	656	NS	Y	.	Y	Y	.	Y	Y[d]	.	.	.	.	.	.	.	.	.
Brugulat-Serrat 2017[39]	575	3.0	Y	.	Y	Y	Y	Y	.	.	.	.	.	.	.	.	.	.
Hoggard 2009[40]	525	1.5 and 3.0	N[h]	.	Y[d]	Y	.	.	.	.	.	.	.	.	.	.	.	Y
Boutet 2016[41]	503	1.5	Y	.	Y	Y	Y			.	.	.		.	.	.	.	.
Kumar 2008[42]	478	1.5	Y	.	Y	.	Y	.	.	.	.	.	.	.	.	.	.	.
Hartwigsen 2010[43]	206	3.0	Y	.	Y	.	Y	.	.	.	.	.	.	.	.	.	.	.
Reneman 2012[44]	203	1.5[i]	N[i]	Y[d]	Y	Y[d]	.	.	.	.	.	.	.	.	.	.	.	Y[d]
Li 2015[45]	167	1.5/3.0[j]	N[j]	.	Y	.	.	.	.	.	.	.	.	.	.	.	.	Y[j]
Illes 2004[46]	151	NS	N[k]	.	Y	Y	.	Y	.	.	.	.	.	.	.	.	.	Y
Wahlund 1989[47]	101	NS	NS[l]	.	.	.	.	.		.	.	.	.	.	.	.	.	.
Menzler 2010[48]	100	1.5	Y	.	Y	Y	Y	.	.	.	.	.	.	.	.	.	.	.
Lubman 2002[49]	98	1.5	N[m]	.	Y	.	.	.	.	.	.	.	.	.	.	.	.	.
Trufyn 2014[50]	56	3.0	Y	.	Y	Y	Y	Y	Y	.	.	.	.	.	.	.	.	Y
**Cardiac MRI only**																		
Vogel Claussen 2009[51]	254	1.5	N[n]	.	.	.	.	.	.	.	.	Y	Y	.	.	.	.	.
Loy 2015[52]	40	3.0	Y	.	.	.	.	.	.	Y	.	Y	.	.	.	.	.	.

FLAIR = fluid attenuated inversion recovery, MRA = magnetic resonance angiography, STIR = short-tau inversion recovery, CE = contrast enhanced, NS = not specified

a. Some studies report the prevalence of incidental findings (IFs) detected in participants who all attended for the same type of imaging, for example, a single research study with a single imaging protocol. These studies are indicated by ‘Y.’ In contrast, some studies report the prevalence of IFs detected in participants scans that have been ‘pooled’ from more than one research project, and therefore involve more than one imaging protocol. These studies are indicated by ‘N,’ with details provided in additional footnotes.
b. Either gradient recalled echo, or susceptibility-weighted imaging.
c. ‘Other’ sequences include additional brain or chest or abdominal sequences, such as proton density, diffusion weighted imaging, colonography, mammography, in and out of phase abdominal imaging etc. Please see individual papers for details.
d. Sequence performed in a subset of participants.
e. Yue 1997:[30] 0.35 Tesla MRI used at three imaging centres, 1.5 Tesla MRI used at one imaging centre. No data available on numbers of participants imaged using the different scanners.
f. Katzman 1999:[35] Participants’ scans were pooled from multiple neuroimaging studies, but all had at least T1- and T2-weighted brain imaging.
g. Sommer 2013:[36] Participants’ scans were pooled from multiple neuroimaging studies, but only T2 images were assessed for IFs.
h. Hoggard 2009:[40] Participants’ scans were pooled from multiple neuroimaging studies, but 456 had at least axial T2-weighted imaging of the whole brain, and the remaining 69 from a single neuroimaging study only had T1-weighted imaging.
i. Reneman 2012:[44] Seven participants were imaged using a 3.0T MRI scanner, with only a 3D T1-weighted sequence.
j. Li 2015:[45] Number of control participants scanned with each magnet was not reported. Participants imaged using the 1.5T scanner underwent 3D-fast spoiled gradient echo T1-weighted imaging. Participants imaged using the 3.0T scanner underwent 3D- magnetization-prepared rapid gradient-echo T1-weighted imaging. Additional, unspecified, sequences were performed when necessary for diagnostic purposes.
k. Illes 2004:[46] Participants’ scans were pooled from multiple neuroimaging studies, and each participant underwent at least one of the sequences indicated.
l. Wahlund 1989:[47] No information on sequence types is available.
m. Lubman 2002:[48] Participants’ scans were pooled from multiple neuroimaging studies, and participants had at least a T1-weighted sequence.
n. Vogel Claussen 2009:[50] Method of imaging coronary arteries differed across the sample: 23 participants underwent steady state free precession coronary MRA images using breath-hold technique, and the remaining 231 underwent 3-dimensional steady-state free precession navigator assisted free breathing whole heart technique.

Supplementary Table 3: 95% prediction intervals (and 95% confidence intervals to enable direct comparison)

IFs and region	Pooled prevalence (%)	95% prediction interval (%)	95 % confidence interval (%)
PSIFs only			
Brain	1.4	0.2 to 8.3	1.0 to 2.1
Thorax	1.3	0 to 76.8	0.2 to 8.1
Abdomen	1.9	0 to 81.1	0.3 to 12.0
Brain and body	3.9	0 to 95.4	0.4 to 27.1
PSIFs and indeterminate IFs			
Brain	1.7	0.2 to 12.3	1.1 to 2.6
Thorax	3.0	0 to 67.4	0.8 to 11.3
Abdomen	4.5	0.2 to 55.2	1.5 to 12.9
Brain and body	12.8	0.4 to 85.7	3.9 to 34.3
Suspected malignant IFs			
Brain	0.6	0.1 to 4.0	0.4 to 0.9
Thorax	0.6	0 to 29.6	0.1 to 3.1
Abdomen	1.3	0 to 76.8	0.2 to 9.3
Brain and body	2.3	0 to 86.5	0.3 to 15.4
Suspected malignant IFs and possible indicators of malignancy			
Brain	0.6	0.1 to 4.2	0.4 to 0.9
Thorax	1.0	0 to 57.4	0.2 to 5.4
Abdomen	1.6	0 to 81.0	0.2 to 10.9
Brain and body	3.0	0 to 91.8	0.4 to 20.4

IFs = incidental findings, PSIFs = potentially serious incidental findings

Supplementary Tables 4a-c: Types of potentially serious incidental findings (PSIFs) by region, in descending order of frequency, as percentages of total PSIFs

Supplementary Table 4a: Brain

Types of PSIFs among 27349 volunteers	N PSIFs	Percentage[a] of PSIFs (total N=688)
Suspected malignancy	317	46
Intracranial mass	179	26
Pituitary mass	67	9.7
Pituitary cyst	55	8.0
Extracranial mass	12	1.7
Atypical cerebellar lesion	2	0.29
Intracranial cyst[b]	1	0.15
Skull: Potentially serious lesion	1	0.15
Suspected aneurysm	214	31
Suspected vascular malformation	106	15
Suspected other:	46	6.7
Arachnoid cyst	18	2.6
Not specified: potentially serious	13	1.9
Acute infarct	4	0.58
Missing pituitary neurohypophysis signal	4	0.58
Subdural haematoma	3	0.44
Colloid cyst	2	0.29
Mesial temporal sclerosis	1	0.15
Syringomyelia	1	0.15
Possible malignancy: hydrocephalus	5	0.73

MRI = magnetic resonance imaging, PSIFs = potentially serious incidental findings
a. All percentages are rounded to two significant figures.
b. This intracranial cyst was followed up with MRI, and we presumed that there were some concerning features, and classified it as a suspected malignancy.

Supplementary Table 4b: Thorax

**Types of PSIFs among 5111 volunteers**	**N PSIFs**	**Percentage[a] of PSIFs (total N=238)**
Suspected malignancy	132	55
Lung	72	30
Nodule	56	24
Lobar pneumonia or lung consolidation	8	3.4
Lesion requiring follow-up	5	2.1
Mass	3	1.3
Other region	60	25
Neck tumor	52	22
Thyroid lesions or enlargement	4	1.7
Cardiac mass	1	0.42
Chest lesion	1	0.42
Liver lesion requiring follow-up	1	0.42
Mediastinal lesion requiring follow-up	1	0.42
Suspected other	55	23
LV hypertrophy	17	7.1
Valve defects	16	6.7
Goitre with tracheal compression	9	3.8
Heart failure	5	2.1
Reduced contractility	5	2.1
Hypertrophic cardiomyopathy	1	0.42
Suspected pulmonary hypertension	1	0.42
Thoracic aortic stenosis	1	0.42
Possible malignancy	41	17
Lymphadenopathy	22	9.2
Pleural effusion	16	6.7
Pericardial effusion	2	0.84
Urinary obstruction	1	0.42
Suspected thoracic aortic aneurysm	10	4.2

MRI = magnetic resonance imaging, PSIFs = potentially serious incidental findings
a. All percentages are rounded to two significant figures.

Supplementary Table 4c: Abdomen

Types of PSIFs among 4817 volunteers	N PSIFs	Percentage[a] of PSIFs (total N=412)
Suspected malignancy	325	79
Renal	131	32
Ovarian	79	19
Liver	50	12
Uterine or cervical malignancy	15	3.6
Pancreas	12	2.9
Adrenal gland	8	1.9
Testicular, epididymal or seminal vesicle	7	1.7
Bladder	6	1.5
Colon or rectum	6	1.5
Spleen	5	1.2
Lumbar intraspinal neurinoma	1	0.24
Lumbar spine lesion requiring follow-up	1	0.24
Prostate	1	0.24
Psoas	1	0.24
Retroperitoneal mass	1	0.24
Stomach	1	0.24
Possible malignancy	55	13
Biliary dilatation	25	6.1
Lymphadenopathy	16	3.9
Splenomegaly	8	1.9
Chronic urinary obstruction	5	1.2
Ascites	1	0.24
Suspected other	18	4.4
Irregular/nodular liver margin	9	2.2
Haemochromatosis	5	1.2
Abdominal aortic stenosis	3	0.73
Reflux nephropathy	1	0.24
Suspected abdominal aortic aneurysm	14	3.4

MRI = magnetic resonance imaging, PSIFs = potentially serious incidental findings
a. All percentages are rounded to two significant figures.
b. Denominator is 1921 women.
c. Denominator is 2896 men.

Supplementary Table 5: Difference in estimates of prevalence of PSIFs between subgroups

Subgroup analysis and region	Prevalence of PSIFs (%) in the reference group	Prevalence of PSIFs (%) in the comparison group	Difference in estimates of prevalence of PSIFs between subgroups, % (95% CI)	p-value
Imaging setting: non-research (reference) versus research (comparison)				
Brain	0.9 (0.6 to 1.2)	1.8 (1.2 to 2.7)	0.9 (0.2 to 2.2)	0.067
Thorax	0.8 (0.01 to 31.4)	2.6 (0.6 to 10.2)	1.8 (-0.7 to 65.9)	0.335
Abdomen	1.2 (0.04 to 27.1)	3.6 (0.6 to 18.5)	2.5 (-1.1 to 63.9)	0.409
Brain and body	3.1 (0.06 to 63.3)	5.4 (0.7 to 32.0)	2.3 (-3.0 to 81.0)	0.428
Blinding of readers to participants' characteristics: Not blinded or not stated (reference) versus blinded (comparison)				
Brain	1.6 (1.0 to 2.5)	1.3 (0.7 to 2.4)	-0.4 (-1.0 to 1.1)	0.337
Thorax	2.4 (0.4 to 12.9)	0.1 (0.0 to 67.9)	-2.2 (-2.4 to 70.0)	0.659
Abdomen	3.3 (0.7 to 14.6)	0.02 (0.0 to 99.8)	-3.3 (-3.3 to 96.5)	0.659
Brain and body	8.8 (2.0 to 31.7)	0.01 (0.0 to 99.9)	-8.8 (-8.8 to 91.1)	0.633
Number of readers: One reader (reference) versus >1 (comparison)				
Brain	1.7 (1.0 to 2.9)	1.4 (0.7 to 2.6)	-0.3 (-1.1 to 1.4)	0.953

PSIFs = potentially serious incidental finding, CI = confidence interval

Supplementary Tables 6a-c: Summary of available data on potential determinants of prevalence of potentially serious incidental findings (PSIFs, as per our definition) or IFs which required follow-up (as per each study's definition), ordered by descending sample size

Supplementary Table 6a: Age

Study	Sample size (n apparently asymptomatic people)	PSIF type	Summary of results[a]	Significant difference between age groups
Tsushima 2005[33]	1113	IFs requiring follow-up[b,c]	Non-significantly higher prevalence in older versus younger participants (60-84 years, 4/192 [2.1%] vs 34-59 years, 11/921 [1.2%], p=0.3[d])	N
Brugulat-Serrat 2017[39]	575	Cavernoma	Non-significantly higher prevalence in older versus younger participants (65-75 years, 4/119 [3.4%] vs 45-54 years, 2/211 [1.0%], p=0.2[d]; 55-65 years, 8/245 [3.3%] vs 45-54 years, 2/211 [1.0%], p=0.1[d])	N
		Intracranial masses	No significant difference in prevalence of pituitary, intraventricular or cerebellar masses in older versus younger participants (Pituitary masses: 45-54 years, 0/211 [0.0%] vs 55-64 years, 2/245 [0.8%, p=0.5[d]] and vs 65-75 years, 0/119 [0.0%, p=NC]; intraventricular masses: 45-54 years, 1/211 [0.5%] vs 55-64 years, 0/245 [0.0%, p=0.5[d]] vs 65-75 years, 0/119 [0.0%, p=1.0[d]]; cerebellar masses: 45-54 years, 0/211 [0.0%] vs 55-64 years, 1/245 [0.4%, p=1.0[d]] and vs 65-75 years, 0/119 [0.0%, p=NC).	N
Hartwigsen 2010[43]	206	IFs requiring follow-up[e,c]	Participants with IFs requiring follow-up were significantly older than those with IFs which did not (p=0.04[f])	Y
Illes 2004[46]	151	IFs requiring follow-up[g,c]	Significantly higher prevalence in older versus younger participants (≥ 60 years, 41/64 [64.1%] vs < 60 years, 30/87 [34.5%], p=0.001])	Y

PSIF = potentially serious incidental finding, N = no, Y = yes, NC = not calculated as zero frequency in both groups.

a. P-values relate to chi-square tests unless otherwise stated.

b. Tsushima 2005:[33] IFs requiring follow-up, as judged by the study's radiologist.
c. Not all IFs requiring follow-up were classed as PSIFs but the distribution of PSIFs between age groups was not possible to calculate from the reported data; in this context, we use 'IFs requiring follow-up' as an approximate proxy for PSIFs.
d. Two-tailed Fisher exact test.
e. Hartwigsen 2010:[43] IFs requiring follow-up, the method of judging this was not reported.
f. From independent samples t-test, reported in the study paper. No numerical data on age of each group (e.g. mean) were reported.
g. Illes 2004:[46] IFs requiring follow-up, as judged by two neuroradiologists.

Supplementary Table 6b: Sex

Study and body region	Sample size (N women)	PSIF type	n women with PSIF[a] (%)	n men with PSIF[a] (%)	p-value[b]
**Brain**					
Bos 2016[29]	5800 (3194)	Cavernoma	18 (0.6)	19 (0.7)	0.5[c]
		Cerebral aneurysm	90 (2.8)	44 (1.7)	0.006[c]
		Pituitary cyst or mass	35 (1.1)	32 (1.2)	0.7[c]
Yue 1997[30]	3672 (2141)	Cavernoma	4 (0.2)	1 (0.1)	0.4
		Pituitary cyst or mass	2 (0.1)	4 (0.3)	0.2
Tsushima 2005[33]	1113 (352)	IFs requiring follow-up[d,e]	5 (1.4)	10 (1.3)	1.0
Haberg 2016[34]	1006 (530)	Arteriovenous malformation	1 (0.2)	0 (0.0)	1.0
		Cavernoma	2 (0.4)	1 (0.2)	0.4
		Cerebral aneurysm	14 (2.6)	5 (1.1)	0.1
		Glioma	1 (0.2)	0 (0.0)	1.0
		Pituitary cyst or mass	2 (0.4)	1 (0.2)	1.0
		Vestibular schwannoma	0 (0.0)	1 (0.2)	0.5
Sandeman 2013[37]	700 (332)	IFs requiring follow-up[f,e]	7 (2.1)	3 (0.8)	0.2
Brugulat-Serrat 2017[39]	575 (348)	Any brain malignancy	15 (4.3)	3 (1.3)	0.1
Kumar 2008[42]	478 (226)	Pituitary cyst or mass	3 (1.3)	1 (0.4)	0.4
Illes 2004[46]	151 (69)	IFs requiring follow-up[g,e]	4 (5.8)	6 (7.3)	0.8
**Brain and body**					
Morin 2009[25]	148 (54)	PSIFs	1 (1.9)	2 (2.1)	0.4

PSIF = potentially serious incidental finding

a. PSIFs, or IFs requiring follow-up, as appropriate.
b. P-values relate to two-tailed Fisher exact tests unless otherwise stated.
c. P-value relates to chi-square test.
d. Tsushima 2005:[33] IFs requiring further evaluation, as judged by the study's radiologist.
e. Not all IFs requiring follow-up were classed as PSIFs but the distribution of PSIFs between women and men was not possible to calculate from the reported data; in this context, we use 'IFs requiring follow-up' as an approximate proxy for PSIFs.
f. Sandeman 2013:[37] IFs requiring further referral, as judged by the study's geriatrician and radiologists.
g. Illes 2004:[46] IFs requiring further evaluation, as judged by two neuroradiologists.

Supplementary Table 6c: Other factors

Study and body region	N patients[a]	N apparently asymptomatic people[b]	PSIFs	Factor	n PSIF[c]/ N with factor (%)	n PSIF[c]/ N without factor (%)	p-value[d]
**Brain**							
Tsushima 2005[33]	0	1113	IFs requiring follow-up[e,f]	Cardiac disease[g]	0/36 (0.0)	15/1077 (1.4)	1.0
				Headache[h]	1/135 (0.7)	14/978 (1.4)	1.0
				Vertigo/dizziness[h]	4/139 (2.9)	11/974 (1.1)	0.1
				Heavy drinking[i]	0/42 (0.0)	15/1071 (1.4)	1.0
				Heavy smoking[j]	1/131 (0.8)	14/982 (1.4)	1.0
				Hypertensive[k]	4/267 (1.5)	11/846 (1.3)	0.8
				Hyperlipidaemia[l]	7/360 (1.9)	8/753 (1.1)	0.3
Sommer 2013[36]	656	722	PSIFs	Psychotic episode[m]	9/656 (1.4)	13/722 (1.8)	0.7
**Thorax**							
Loy 2015[52]	169	40	IF requiring follow-up[n]	HIV positive	12/169 (7.1)	1/40 (2.5)	0.5
**Brain and body**							
Saya 2017[27]	44	44	Malignancy	TP53 mutation	4/44 (9.1)	0/44 (0.0)	0.1

PSIF = potentially serious incidental findings, - cannot be calculated, HIV = human immunodeficiency virus, NR = not reported

a. Some studies included in the review involved patient groups, data from these were not included in the review, but are presented here to summarise prevalences of IFs between patient groups and apparently asymptomatic people.
b. Corresponds to sample sizes reported elsewhere in this review, i.e. apparently asymptomatic people only.
c. PSIFs, or IFs requiring follow-up, as appropriate.
d. P-values relate to two-tailed Fisher exact tests.
e. Tsushima 2005:[33] IFs requiring further evaluation, as judged by the study's radiologist.
f. Not all IFs requiring follow-up were classed as PSIFs but the distribution of PSIFs between groups with and without each factor was not possible to calculate from the reported data; in this context, we use 'IFs requiring follow-up' as an approximate proxy for PSIFs.
g. Cardiac disease included congestive heart failure, myocardial infarction, angina pectoris, left ventricular hypertrophy atrial fibrillation; or electrocardiographic evidence of past myocardial infarction, left ventricular hypertrophy, or atrial fibrillation.
h. From a medical history taken by a neurologist.
i. ≥ 60g of alcohol per day.
j. > 20 cigarettes per day.
k. Systolic blood pressure > 140 mmHg, or diastolic blood pressure > 90 mmHg, or on treatment for hypertension, or history of hypertension.
l. Fasting total cholesterol > 250 mg/dl, or history of hyperlipidaemia.
m. Sommer 2013:[36] Not further defined.
n. Loy 2015:[52] IFs requiring follow-up, not further described.
